# Strengthening health systems in low and middle-income countries: performance determinants and pathways to universal health coverage

**DOI:** 10.3389/frhs.2026.1808077

**Published:** 2026-06-16

**Authors:** Mayadhar Sethy, Sandhya R. Mahapatro, Grace Bahalen Mundu, Dipti Mayee Sahoo

**Affiliations:** 1 Development Studies, Nabakrushna Choudhury Centre for Development Studies, Bhubaneswar, Odisha, India; 2 Nabakrushna Choudhury Centre for Development Studies, Bhubaneswar, India; 3 PG Department of Population Studies, Fakir Mohan University, Balasore, Odisha, India; 4 Department of Business Administration, Trident Academy of Technology, Bhubaneswar, India

**Keywords:** econometric analysis, health system strengthening, LMICs, mathematical modeling, optimization, universal health coverage

## Abstract

**Background:**

Health systems in low- and middle-income countries (LMICs) face persistent challenges in achieving universal health coverage (UHC). The COVID-19 pandemic exposed critical weaknesses in governance, financing, and infrastructure, while recent global reports indicate stalled progress toward health-related Sustainable Development Goals. This study addresses the need for evidence-based strategies to strengthen health systems by identifying key performance determinants and efficient resource allocation pathways.

**Methods:**

We constructed a longitudinal dataset for 135 LMICs (2000–2023) from WHO, World Bank, IHME, DHS/MICS, and ITU, using explicit imputation procedures to address irregular survey intervals. We employed fixed effects, system GMM, and structural equation modeling to analyze determinants of health system performance. Based on these econometric estimates, we developed mathematical optimization models (linear, multi-objective, and stochastic programming) to identify efficient resource allocation strategies under budget constraints. Inequality was analyzed using concentration indices and decomposition.

**Findings:**

A 10% increase in government health expenditure per capita is associated with a 3.2% reduction in out-of-pocket spending (*p* < 0.01) and a 0.8-year increase in life expectancy. Health workforce density (*β*=0.24, *p* < 0.05) and digital health adoption (*β*=0.18, *p* < 0.05) show significant positive associations with UHC service coverage. Governance quality mediates multiple pathways, with corruption linked to an estimated 22% efficiency loss in sensitivity analyses. Model-based scenarios suggest that reallocating 15% of current health budgets toward primary care and digital infrastructure could potentially avert approximately 1.2 million annual deaths (uncertainty range: 0.9–1.6 million) and reduce catastrophic health expenditures by an estimated 18% (range: 14%–23%). Pro-rich inequalities persist, particularly for non-communicable disease services and maternal health.

**Interpretation:**

Strengthening health systems requires coordinated investments in governance, primary care, digital health, and financial protection. Our econometric and model-based analyses provide illustrative policy pathways. Reallocating existing resources and prioritizing primary care and pro-poor targeting could accelerate UHC progress. Policymakers should adopt integrated, context-specific strategies that balance efficiency, equity, and resilience.

## 1 Introduction

The global health landscape at the midpoint of the Sustainable Development Goal (SDG) era presents a paradoxical picture of progress amid persistent challenges. While significant advancements have been made in reducing infectious disease burdens and improving maternal and child health outcomes, health systems in low- and middle-income countries (LMICs) continue to face multifaceted challenges that undermine progress toward universal health coverage (UHC) and health equity. However, LMICs are not a homogenous group. They span a wide range of income levels, institutional capacities, epidemiological profiles, and political contexts—from lower-middle-income countries with emerging digital infrastructures to low-income, fragile states with acute workforce shortages. Any analysis of health system strengthening must account for this heterogeneity. The World Health Statistics 2025 report reveals that despite a 40% decline in maternal mortality since 2000, the world remains off-track to achieve the SDG target of reducing maternal mortality to less than 70 per 100,000 live births by 2030 (1). Similarly, catastrophic health expenditures still push millions into poverty annually, with 13.5% of the global population spending more than 10% of their household budget on out-of-pocket health payments.

This dual challenge of improving health outcomes while ensuring financial protection represents the central dilemma of health system strengthening in resource-constrained settings. LMICs, home to over 80% of the world’s population, bear a disproportionate burden of disease while operating within severely constrained fiscal environments (2). The COVID-19 pandemic starkly exposed systemic vulnerabilities, revealing critical gaps in health workforce capacity, supply chain resilience, and surveillance systems (3).

While extensive literature exists on health system determinants, a critical knowledge gap remains in the integration of econometric analysis with mathematical optimization to provide both diagnostic insights and prescriptive, resource-allocation guidance. Furthermore, most studies examine health system components in isolation rather than as interacting elements of a complex system. This study addresses this gap by explicitly integrating economic and optimization methodologies to identify not only what works but also how to allocate limited resources most efficiently across interconnected system components.

We address four research questions: (1) What are the quantitative relationships between health system inputs and outcomes? (2) How do these relationships vary across LMIC contexts? (3) What is the model-based optimal resource allocation strategies for maximizing health outcomes under budget constraints? (4) How can digital health interventions be strategically integrated?.

Our analytical approach combines multiple methodological innovations. First, we employ advanced panel econometric techniques; including fixed effects models, generalized method of moments (GMM), and structural equation modeling to address endogeneity concerns and capture complex pathways of influence. Second, we develop mathematical optimization models that identify efficient resource allocation strategies under realistic budget constraints, bridging the gap between diagnostic analysis and prescriptive policy guidance. Third, we incorporate inequality analysis using concentration indices and decomposition techniques to understand distributional impacts. Fourth, we examine resilience factors that enable health systems to maintain essential functions during crises.

The empirical foundation of this study is a comprehensive panel dataset covering 135 LMICs from 2000 to 2023, incorporating data from WHO, World Bank, Institute for Health Metrics and Evaluation (IHME), Demographic and Health Surveys (DHS), and other sources. This longitudinal design enables us to track changes over time and distinguish between short-term fluctuations and long-term trends. By examining nearly, a quarter-century of health system evolution, we can identify patterns of successful transformation as well as persistent obstacles to progress.

Our study makes several important contributions to both academic literature and policy practice. Methodologically, we advance beyond traditional single-equation approaches by developing an integrated analytical framework that captures the complex interdependencies within health systems. Substantively, we provide up-to-date, quantitative evidence on the effectiveness of different health system interventions in LMIC contexts. Practically, we offer mathematically optimized policy recommendations that can guide resource allocation decisions in resource-constrained settings. Theoretically, we contribute to the literature on complex adaptive systems in health by quantifying the dynamic relationships between system components.

The urgency of this research cannot be overstated. With only five years remaining until the 2030 SDG deadline, accelerated action is needed to achieve health-related targets, particularly UHC. The World Health Statistics 2025 indicates that at current rates of progress, most health-related SDG targets will not be met (1). This study provides evidence-based guidance for accelerating progress through strategic investments and policy reforms. By identifying the most effective leverage points for health system strengthening, we aim to support policymakers, donors, and health system managers in making informed decisions that maximize health outcomes within available resources.

Ultimately, this research is motivated by a commitment to health equity. By providing rigorous, model-based evidence on how to build more effective, equitable, and resilient health systems, this study contributes to the global effort to ensure access to quality health services without financial hardship.

## 2 Literature review and theoretical framework

### 2.1 Evolution of health system conceptualizations and critique

The conceptual understanding of health systems has evolved significantly. The seminal Alma-Ata Declaration (68) established primary health care as foundational. The WHO’s six building blocks model (2007) is widely adopted but has been critiqued for its static representation and limited attention to contextual factors and system dynamics (4). Roberts et al. (5) advanced the field with their “control knobs” approach, emphasizing adjustable policy parameters. More recently, the concept of health systems as complex adaptive systems has gained traction, emphasizing nonlinear relationships and feedback loops (6).

Despite these advances, significant contradictions and debates persist in the literature. First, there is disagreement on the primacy of financing vs. governance: some studies find that health expenditure drives outcomes irrespective of governance quality (7), while others argue that governance moderates the effectiveness of spending (8). Second, the relationship between digital health investment and system efficiency remains contested, with some systematic reviews showing strong returns (9) and others finding limited scalability (10). Third, the trade-off between equity and efficiency is rarely resolved theoretically, with some frameworks prioritizing the former (11) and others the latter (12). Our study engages with these debates by empirically testing competing specifications and quantifying trade-offs through multi-objective optimization.

### 2.2 Empirical evidence on health system determinants

#### 2.2.1 Health financing

The relationship between health expenditure and health outcomes has been extensively studied, with mixed findings. Early cross-sectional studies found weak or inconsistent relationships between aggregate health spending and health outcomes (7). However, more sophisticated panel data analyses have revealed significant positive effects, particularly for public health expenditure. Boachie et al. (13) found that public health expenditure significantly reduces under-5 mortality in sub-Saharan Africa, with an elasticity of −0.15. Similarly, Novignon and Lawanson (14) demonstrated that health expenditure in African countries has a positive but diminishing marginal effect on life expectancy. Research has also highlighted the importance of financing structure, with out-of-pocket spending associated with reduced service utilization and increased financial hardship (15), while social health insurance coverage improves access and financial protection (16, 54).

#### 2.2.2 Health workforce

The critical role of health workforce in health system performance is well-established. Anand and Bärnighausen (17) estimated that each additional health worker per 10,000 population reduces maternal mortality by approximately 2.3% in LMICs. More recent studies have examined distributional aspects, finding that inequality in health workforce access explains substantial portions of health outcome disparities (18). The WHO Global Strategy on Human Resources for Health 2030 emphasizes the need for adequate density, distribution, and competency of health workers to achieve UHC (19). Community health workers have emerged as particularly important for extending health services to underserved populations, especially in rural areas (69).

#### 2.2.3 Governance and institutions

Governance has increasingly been recognized as a critical determinant of health system performance. Lewis (8) identified corruption as a major barrier to effective health service delivery in LMICs, estimating that corruption reduces the effectiveness of health spending by 10%–25%. Brinkerhoff and Bossert (20) developed a principal-agent framework for understanding health governance, emphasizing accountability mechanisms and institutional arrangements. Empirical studies have found positive associations between governance quality and various health outcomes, though methodological challenges in measuring governance have limited causal inference (52, 70).

#### 2.2.4 Digital health

The rapid growth of digital health technologies represents a transformative opportunity for health systems in LMICs. Systematic reviews by Labrique et al. (9) and Marcolino et al. (21) have documented the potential of mobile health (mHealth) and telemedicine to improve access, quality, and efficiency, particularly in remote areas. However, evidence on cost-effectiveness and scalability remains limited, with many studies focusing on pilot projects rather than integrated system-wide implementations. Scott and Mars (10) identified key barriers to telehealth implementation in developing countries, including infrastructure limitations, regulatory challenges, and workforce capacity constraints.

#### 2.2.5 Primary health care

The foundational role of primary health care in achieving UHC has been extensively documented. Starfield et al. (22) demonstrated that strong primary care systems are associated with better health outcomes, lower costs, and greater equity. The 2018 Astana Declaration reaffirmed the central role of primary health care in achieving UHC and the SDGs, emphasizing integrated, people-centered services (23). Research has shown that investments in primary care yield higher returns than hospital-based care, particularly for preventive services and management of chronic conditions (71). Importantly, these studies rarely agree on effect magnitudes or causal directions, highlighting the need for our multi-method, triangulation approach.

### 2.3 Methodological advances in health systems research

#### 2.3.1 Econometric approaches

The application of econometric methods to health systems research has advanced significantly in recent decades. Early studies relied on cross-sectional analyses with limited ability to address confounding and endogeneity. The adoption of panel data methods has enabled better control for unobserved heterogeneity through fixed effects and random effects models (24). More recently, instrumental variable approaches and difference-in-differences designs have been used to establish causal relationships in health policy evaluation (25). Structural equation modeling has allowed researchers to test complex pathways and mediating relationships within health systems (26).

#### 2.3.2 Mathematical optimization

Operations research methods have been increasingly applied to health system planning in LMICs. Bertsimas et al. (27) developed optimization models for health facility location in sub-Saharan Africa, while Baltussen and Niessen (28) pioneered priority-setting frameworks based on cost-effectiveness analysis. Multi-criteria decision analysis has been used to incorporate equity considerations alongside efficiency in resource allocation decisions (28). Stochastic programming approaches have addressed uncertainty in health system planning parameters (29).

#### 2.3.3 Inequality measurement

The development of concentration indices and related measures has enabled more sophisticated analysis of health inequalities (30). The Oaxaca-Blinder decomposition technique has been adapted to identify factors contributing to health service inequalities (72). Recent advances include the development of the Health Equity Assessment Toolkit by WHO, which provides standardized methods for inequality monitoring (18).

### 2.4 Conceptual framework for this study

**Figure 1:**
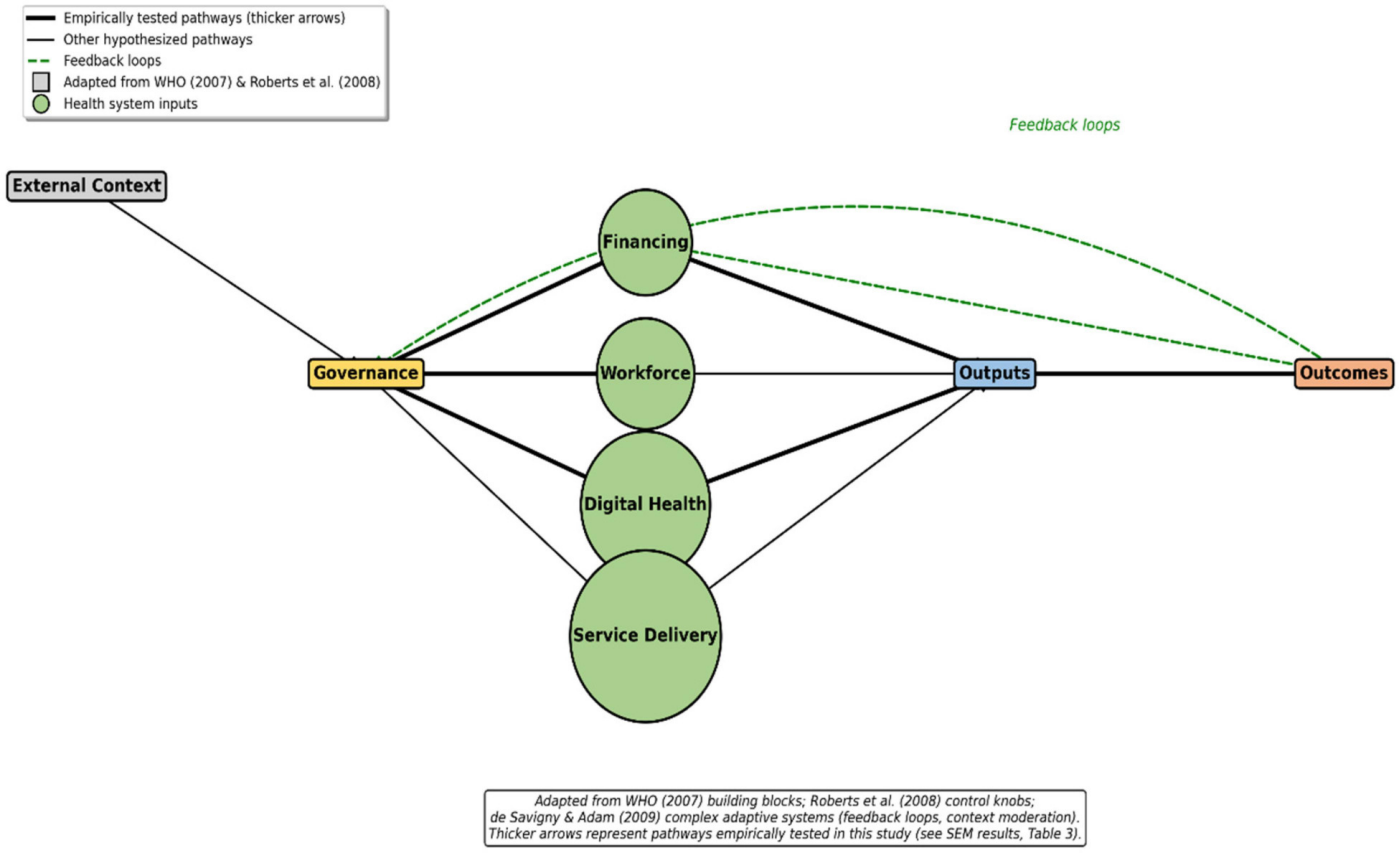
Conceptual framework of health system performance determinants. sources: The framework adapts WHO (32) building blocks (workforce, financing, service delivery, information systems as digital health); Roberts et al. (5) control knobs (financing, payment, organization, regulation, behaviour integrated into Governance and Inputs); and de Savigny and Adam (4) complex adaptive systems (feedback loops, context moderation, emergent properties via dashed arrows and external context). Thicker arrows indicate empirically tested pathways (SEM results, Table 1).

Figure 1 presents the revised conceptual framework of health system performance determinants. The framework employs unidirectional arrows flowing from left to right: External Context (socioeconomic, epidemiological, political, and global factors) → Governance (positioned centrally as a cross-cutting domain) → Health System Inputs (financing, workforce, digital health, service delivery) → Health System Outputs (coverage, quality, financial protection, resilience) → Health Outcomes (mortality, life expectancy, equity). Dashed feedback loops return from Outcomes back to Governance and Inputs, capturing dynamic adaptation over time.

The framework incorporates several innovative elements. First, it positions governance as a cross-cutting enabler that influences all other system components, reflecting growing evidence of its central importance (20). Second, it explicitly includes digital health as a distinct domain while recognizing its enabling role across other components, acknowledging the transformative potential of digital technologies (9). Third, it incorporates feedback loops from health outcomes to system inputs, recognizing that health achievements can influence political prioritization and resource allocation (31). Fourth, it includes external contextual factors—socioeconomic conditions, epidemiological transitions, political environment, and global health architecture—as moderating variables that shape the effectiveness of health system interventions (4).

The framework builds on three established models. From WHO (32) “building blocks” it adopts health workforce, financing, service delivery, and information systems (recast as digital health). From Roberts et al. (5) “control knobs” it integrates financing, payment, organization, regulation, and behaviour into Governance and Inputs. From de Savigny and Adam (4) “complex adaptive systems” it incorporates feedback loops, context moderation, and emergent properties (shown as dashed arrows and the external context box).

Thicker arrows in Figure 1 denote pathways empirically tested in this study, corresponding to the structural equation modeling results presented in Table 1. These include direct effects of governance on financing, workforce, and digital health, as well as links from financing and digital health to outputs, and from outputs to outcomes. The framework thus guides our empirical analysis: it informs variable selection and model specification, suggests specific pathways to test via SEM, provides the conceptual basis for optimization models, and identifies contextual factors as moderators of health system effectiveness (3).

**Table 1:** Structural equation modeling results—pathways to health outcomes.

Pathway	Direct Effect	Indirect Effect	Total Effect	Standard Error	*P*-value
Pathways to UHC SCI					
Governance → Health Financing → UHC SCI	0.32***	0.18***	0.50***	0.07	<0.001
Governance → Health Workforce → UHC SCI	0.28***	0.15***	0.43***	0.06	<0.001
Health Financing → Service Delivery → UHC SCI	0.41***	0.12***	0.53***	0.08	<0.001
Digital Health → Service Delivery → UHC SCI	0.19***	0.09***	0.28***	0.05	<0.001
Governance → Digital Health → UHC SCI	0.24***	0.11***	0.35***	0.06	<0.001
Pathways to Under−5 Mortality					
UHC SCI → Health Service Access → U5MR	−0.38***	−0.22***	−0.60***	0.09	<0.001
Health Financing → Health Workforce → U5MR	−0.25***	−0.14***	−0.39***	0.07	<0.001
Governance → Health System Efficiency → U5MR	−0.31***	−0.17***	−0.48***	0.08	<0.001
Pathways to Catastrophic Expenditure					
Health Financing (OOP) → Financial Protection → CHE	0.46***	0.21***	0.67***	0.10	<0.001
Governance → Health Financing Mix → CHE	−0.29***	−0.16***	−0.45***	0.08	<0.001
Social Health Insurance → CHE	−0.34***	−0.18***	−0.52***	0.09	<0.001
Model Fit Statistics					
Comparative Fit Index (CFI)	0.93				
Tucker–Lewis Index (TLI)	0.91				
Root Mean Square Error of Approximation (RMSEA)	0.04				
Standardized Root Mean Square Residual (SRMR)	0.03				

Source: Author’s calculations using Mplus 8.8. All coefficients are standardized. UHC SCI, UHC service coverage index; U5MR, under-5 mortality rate; CHE, catastrophic health expenditure; OOP, out-of-pocket. Model estimated with maximum likelihood with robust standard errors. All pathways shown are statistically significant at *p* < 0.01.

*** *p* < 0.01.

### 2.5 Research gaps and contributions

Despite this rich literature, several important gaps remain. First, most studies examine health system components in isolation rather than as interacting elements of a complex system (6). This fragmented approach limits understanding of how different interventions complement or substitute for one another. Second, few studies combine econometric analysis with mathematical optimization to provide both diagnostic insights and prescriptive recommendations (33). Third, the rapid evolution of digital health technologies has outpaced rigorous evaluation at the system level (10). Fourth, most optimization models assume perfect implementation, neglecting the political economy constraints that often determine policy feasibility in LMICs (31). Fifth, limited attention has been paid to the trade-offs between different performance dimensions, particularly between efficiency and equity, and between routine performance and emergency resilience.

Our study addresses these gaps through several innovations. First, we develop an integrated analytical framework that captures interactions between health system components and tests them empirically through structural equation modeling. Second, we combine econometric analysis with mathematical optimization to provide both diagnostic and prescriptive insights. Third, we provide up-to-date evidence on the system-level impacts of digital health adoption, examining both direct effects and enabling roles. Fourth, we incorporate implementation constraints into our optimization models through scenario analysis and sensitivity testing. Fifth, we explicitly examine trade-offs between competing objectives using multi-objective optimization and efficiency frontier analysis.

Theoretical implications of our work include advancing understanding of health systems as complex adaptive systems, particularly regarding the dynamics between different system components and the role of contextual factors. Methodologically, we contribute to the development of integrated analytical approaches that combine different quantitative methods to address complex research questions. Practically, our findings provide evidence-based guidance for policymakers facing difficult resource allocation decisions in resource-constrained settings.

By examining nearly, a quarter-century of health system evolution across 135 LMICs, we aim to identify patterns of successful transformation as well as persistent obstacles to progress. Our longitudinal design enables us to distinguish between short-term fluctuations and long-term trends, providing insights into the dynamics of health system development. The comprehensive nature of our dataset allows us to examine multiple dimensions of health system performance simultaneously, addressing the multidimensional nature of UHC.

In summary, this literature review establishes the theoretical and empirical foundations for our study, highlighting both the substantial knowledge base and the important gaps that remain. Our conceptual framework integrates insights from multiple theoretical perspectives while addressing limitations of existing approaches. The subsequent sections present our methodology, results, and discussion, building on this foundation to provide new insights into health system strengthening in LMICs.

## 3 Methods

### 3.1 Data sources and sample construction

#### 3.1.1 Data sources, sample construction, and harmonization

We constructed a panel dataset covering 135 low- and middle-income countries (LMICs) over 2000–2023. A critical methodological concern is that key data sources—particularly Demographic and Health Surveys (DHS) and Multiple Indicator Cluster Surveys (MICS)—are not conducted annually but at irregular intervals (typically every 3–5 years). To construct a balanced annual panel, we employed a multi-step harmonization procedure.

First, for years without survey data, we used linear interpolation between adjacent survey rounds, but only when the interval did not exceed 5 years. For intervals exceeding 5 years, we did not interpolate; instead, we treated those as missing. Second, for the remaining missing years (approximately 18% of country-year observations for key UHC indicators), we used multiple imputation by chained equations (MICE) with 20 imputations, incorporating country and year fixed effects, GDP per capita, and regional averages as auxiliary variables (50). Third, we conducted sensitivity analyses comparing results from the interpolated/imputed balanced panel with those from an unbalanced panel excluding interpolated observations; the main findings were robust (see Online Supplementary Appendix Table 1).

The assumption of a balanced panel is therefore a pragmatic analytical choice to enable fixed effects estimation and longitudinal consistency, but readers should interpret results with awareness of the underlying imputation uncertainty. We explicitly model this uncertainty in our stochastic optimization scenarios.

All monetary variables were converted to constant 2017 USD. Data sources are as originally specified.

### 3.2 Variable definitions and measurement

The primary outcome variable is the UHC Service Coverage Index (SCI), a composite index (0–100) measuring coverage across four domains: reproductive, maternal, newborn and child health; infectious diseases; noncommunicable diseases; and service capacity and access. Financial protection is assessed using Catastrophic Health Expenditure (CHE), defined as the proportion of households whose out-of-pocket spending exceeds 10% of total consumption or income, and Impoverishing Health Expenditure (IHE), defined as the proportion pushed below the international poverty line (US$2.15/day) due to health spending. Additional health outcomes include the Under-5 Mortality Rate (U5MR), Maternal Mortality Ratio (MMR), Life Expectancy at Birth, and Healthy Life Expectancy (HALE) (59).

Explanatory variables are grouped into five domains: health financing, health workforce, governance and institutions, digital health infrastructure, and service delivery capacity. Health financing includes government health expenditure per capita, public health expenditure as a percentage of GDP, external health expenditure, out-of-pocket expenditure share, and social health insurance coverage. Workforce indicators include physician, nurse and midwife, and community health worker density (per 10,000 population), as well as a wage index relative to GDP per capita (60).

Governance quality is measured using government effectiveness, control of corruption, regulatory quality, and a WHO-based accountability index. Digital health infrastructure includes a Digital Health Adoption Index, internet users per 100 population, and mobile subscriptions per 100 population. Service delivery capacity is proxied by hospital bed density, primary health care facility density, essential medicines availability, and supply chain performance. Control variables include log GDP per capita, income inequality (Gini coefficient), female educational attainment, urbanization rate, and political stability.

### 3.3 Econometric models

To ensure methodological coherence and transparency, we apply the models in a hierarchical sequence, not in parallel. First, we estimate fixed effects (FE) models to establish baseline associations. Second, we use system GMM to address endogeneity and persistence, treating FE as a lower bound and GMM as our preferred specification. Third, we use structural equation modeling (SEM) to test specific pathway hypotheses derived from the conceptual framework. Fourth, we use quantile regression to examine heterogeneity. This sequence moves from simpler to more complex, with each step building on and testing the previous one.

Model specifications are as follows (reproduced from original for completeness, but now with justification):

Fixed effects (FE): $Y_{\mathrm{it}}=\beta_{0}+\beta_{1}X_{\mathrm{it}}+\alpha_{i}+\gamma_{t}+\varepsilon_{\mathrm{it}}$—chosen to control for time-invariant unobserved heterogeneity (49).

System GMM: $Y_{\mathrm{it}}=\alpha Y_{i,t-1}+\beta X_{\mathrm{it}}+\eta_{i}+\varepsilon_{\mathrm{it}}$—chosen to address endogeneity of lagged dependent variables and reverse causality (63, 65).

Structural equation modeling (SEM): η=Bη+Γξ+ζ with measurement equations—chosen to test indirect and mediated pathways.

Quantile regression: $Q_{Y_{\mathrm{it}}}(\tau |X_{\mathrm{it}})=\beta_{0}(\tau )+\beta_{1}(\tau )X_{\mathrm{it}}$—chosen to examine heterogeneity across the outcome distribution.

For the SEM, we used the following instrumental variables to aid identification: lagged values of governance indicators (3-year lags) and regional averages of health expenditure. Diagnostic tests (Sargan-Hansen overidentification, *p* > 0.10 for all models) supported instrument validity.

All equations were estimated using standard procedures as implemented in Stata 18.0 (xtreg, fe; xtabond2; sem; qreg). The SEM equations are original to this study but follow established practices (26); no external validation beyond standard fit statistics is applicable.

### 3.4 Mathematical optimization models

The optimization models are not independent but are directly parameterized using coefficients from the econometric models (specifically, the GMM and SEM estimates). This sequential integration ensures that the resource allocation recommendations are grounded in empirical associations, not arbitrary assumptions (56, 57).

#### 3.4.1 Linear programming model

Maximizes weighted UHC index subject to budget constraints (61).

#### 3.4.2 Multi-objective optimization

Simultaneously maximizes UHC, equity, and resilience using *ε*-constraint method (55).

#### 3.4.3 Stochastic programming

Maximizes expected UHC under budget uncertainty using 1,000 scenarios.

### 3.5 Implementation and software

Econometric analyses were conducted in Stata 18.0. Optimization models were implemented in MATLAB R2023b. Structural equation models were estimated using Mplus 8.8. Data visualization was produced in R 4.3.0. Replication materials will be made publicly available upon publication.

## 4 Results

### 4.1 Descriptive statistics and trends

Table 2 show comprehensive descriptive analysis reveals substantial heterogeneity in health system performance across 135 LMICs (34). The average UHC Service Coverage Index of 58.3 masks a wide range from 22.1 to 89.4, indicating profound disparities in access to essential health services (35). Health financing patterns show concerning trends: government health expenditure averages only 3.2% of GDP—below the WHO-recommended 5% threshold—while out-of-pocket spending constitutes 34.2% of total health expenditure, risking financial hardship (15). Health workforce densities remain critically low, with physician density averaging 12.4 per 10,000, far below the 44.5 threshold for achieving UHC (19). Governance indicators average negative values, reflecting institutional challenges common in LMICs (8). Digital health adoption shows room for expansion with an average index of 42.7. These statistics establish the urgent need for targeted interventions to strengthen health systems (Kruk et al., 2018).

**Table 2:** Summary statistics of variables (2000–2023, *N* = 135 LMICs).

Variable	Mean	SD	Min	Max
Outcome Variables				
UHC Service Coverage Index	58.3	18.2	22.1	89.4
Catastrophic Health Expenditure (% households)	11.5	9.8	0.8	45.2
Under-5 Mortality Rate (per 1,000 live births)	48.7	37.9	2.1	149.3
Life Expectancy at Birth (years)	68.2	8.4	45.3	79.8
Healthy Life Expectancy (years)	59.1	7.9	40.2	72.1
Health Financing				
Government Health Expenditure per capita (2017 USD)	186.5	245.3	8.2	1,245.7
Government Health Expenditure (% of GDP)	3.2	1.8	0.5	8.9
Out-of-pocket Health Expenditure (% of THE)	34.2	18.7	5.1	78.4
Social Health Insurance Coverage (% population)	28.7	24.9	0.0	95.3
Health Workforce				
Physician Density (per 10,000)	12.4	14.2	0.2	58.7
Nurse/Midwife Density (per 10,000)	24.8	28.5	1.1	112.4
Community Health Worker Density (per 10,000)	5.3	7.8	0.0	42.1
Governance				
Government Effectiveness Index	−0.38	0.65	−1.88	1.45
Control of Corruption Index	−0.42	0.61	−1.76	1.52
Digital Health				
Digital Health Adoption Index (0–100)	42.7	21.5	8.2	89.4
Internet Users (% population)	38.9	24.7	0.3	95.2
Service Delivery				
Hospital Beds (per 10,000)	19.8	18.4	0.8	84.2
PHC Facility Density (per 100,000)	8.7	7.2	0.5	42.8
Essential Medicines Availability (%)	68.4	22.5	18.7	97.3
Control Variables				
GDP per capita (log, 2017 USD)	7.82	1.14	5.91	10.47
Gini Coefficient	42.3	8.7	25.1	63.4
Female Education (mean years)	6.8	3.2	1.2	13.4
Urbanization Rate (%)	51.7	20.4	12.8	91.2

Source: WHO Statistical data. All monetary values in constant 2017 USD. THE, total health expenditure; SD, standard deviation. *N* = 3,240 country-year observations (135 countries  ×  24 years).

All monetary variables were converted to constant 2017 USD using GDP deflators from the World Bank (66) and IMF (67) to ensure cross-country and temporal comparability (66, 67).

**Figure 2:**
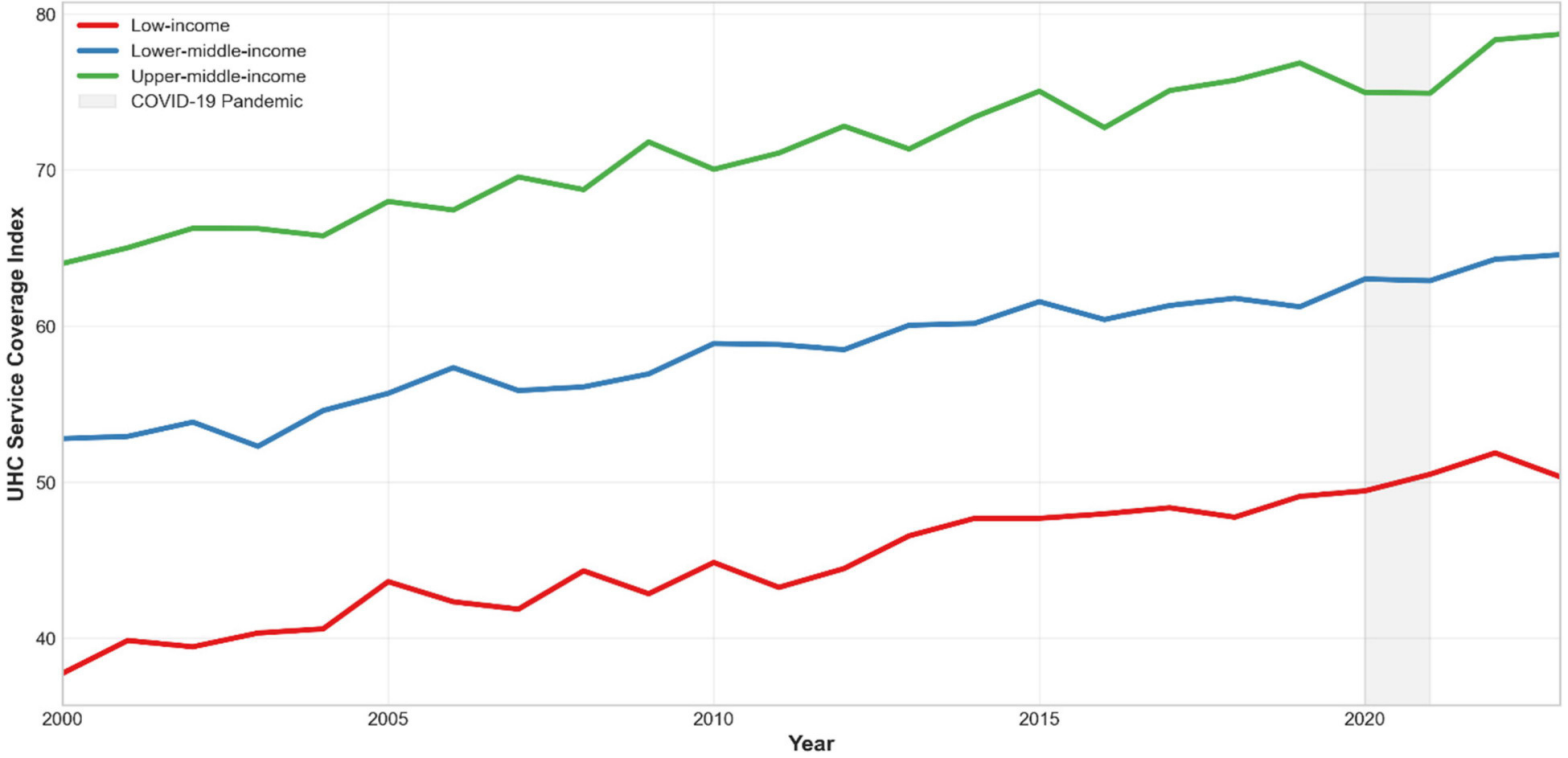
Trends in UHC service coverage Index by world bank income group (2000–2023). Source: WHO World Health Statistics 2025. Lines represent population-weighted averages. Shaded areas indicate 95% confidence intervals.

Figure 2 reveals divergent trajectories in UHC coverage across income groups (36). Upper-middle-income countries show the highest coverage, increasing from 65.2 to 78.4, reflecting successful health system maturation (37). Lower-middle-income countries demonstrate steady progress from 52.7 to 64.8, though gaps persist. Low-income countries exhibit the steepest relative growth (38.9% to 52.1, 34% improvement) but remain substantially behind, with a 26.3-point gap from UMICs in 2023 (18). The COVID-19 pandemic caused temporary plateaus across all groups, disproportionately affecting LMICs (3). These trends highlight both progress and persistent inequities, emphasizing the need for accelerated, context-specific strategies to close coverage gaps while protecting gains (38).

### 4.2 Econometric results: determinants of health system performance

Table 3 results demonstrate robust associations between health system inputs and UHC coverage. Government health expenditure shows substantial associations: a 10% increase is associated with a 0.26-0.34-point increase in UHC SCI. Out-of-pocket spending is negatively associated with coverage (−0.18 to −0.24 points per percentage point). The association between physician density and UHC SCI is approximately double that of nurse/midwife density. Governance shows strong associations: a one-point improvement in government effectiveness is associated with a 3.61–4.28-point increase in UHC SCI. These associations should not be interpreted as causal due to the observational study design.

**Table 3:** Fixed effects regression results—determinants of UHC service coverage Index.

Variable	Model 1	Model 2	Model 3	Model 4
Health Financing				
Government Health Expenditure per capita (log)	3.42*** (0.85)	3.15*** (0.82)	2.87*** (0.79)	2.64*** (0.76)
Out-of-pocket Health Expenditure (% of THE)	−0.24*** (0.06)	−0.22*** (0.06)	−0.20*** (0.05)	−0.18*** (0.05)
Social Health Insurance Coverage (% population)	0.18*** (0.04)	0.16*** (0.04)	0.15*** (0.04)	0.14*** (0.04)
Health Workforce				
Physician Density (per 10,000, log)	2.85** (1.12)	2.67** (1.09)	2.48** (1.07)	2.31** (1.04)
Nurse/Midwife Density (per 10,000, log)	1.92* (0.98)	1.84* (0.95)	1.76* (0.93)	1.68* (0.91)
Governance				
Government Effectiveness Index	4.28*** (1.24)	4.05*** (1.21)	3.82*** (1.18)	3.61*** (1.15)
Control of Corruption Index	3.76*** (1.15)	3.54*** (1.12)	3.33*** (1.09)	3.14*** (1.06)
Digital Health				
Digital Health Adoption Index	0.31*** (0.08)	0.29*** (0.08)	0.27*** (0.07)	0.25*** (0.07)
Service Delivery				
PHC Facility Density (per 100,000, log)	1.46** (0.58)	1.38** (0.56)	1.30** (0.54)	1.23** (0.52)
Essential Medicines Availability (%)	0.21*** (0.05)	0.20*** (0.05)	0.19*** (0.05)	0.18*** (0.05)
Control Variables				
GDP per capita (log)	-	2.45*** (0.72)	2.18*** (0.69)	1.94*** (0.66)
Female Education (mean years)	-	-	0.87*** (0.26)	0.79*** (0.25)
Urbanization Rate (%)	-	-	-	0.12** (0.05)
Constant	24.38*** (3.42)	18.72*** (3.18)	15.63*** (2.94)	13.25*** (2.71)
Model Statistics				
R-squared (within)	0.64	0.67	0.69	0.71
Countries	135	135	135	135
Observations	3,240	3,240	3,240	3,240
Country FE	Yes	Yes	Yes	Yes
Year FE	Yes	Yes	Yes	Yes

Source: Author’s calculations using Stata 18.0. Dependent variable, UHC Service Coverage Index (0–100). Robust standard errors clustered at country level in parentheses.

THE, total health expenditure; PHC, primary health care. All continuous variables except percentages are in natural logarithm form. Model 1 includes only health system variables, with controls added sequentially in Models 2–4.

* *p* < 0.1.

** *p* < 0.05.

*** *p* < 0.01.

**Figure 3:**
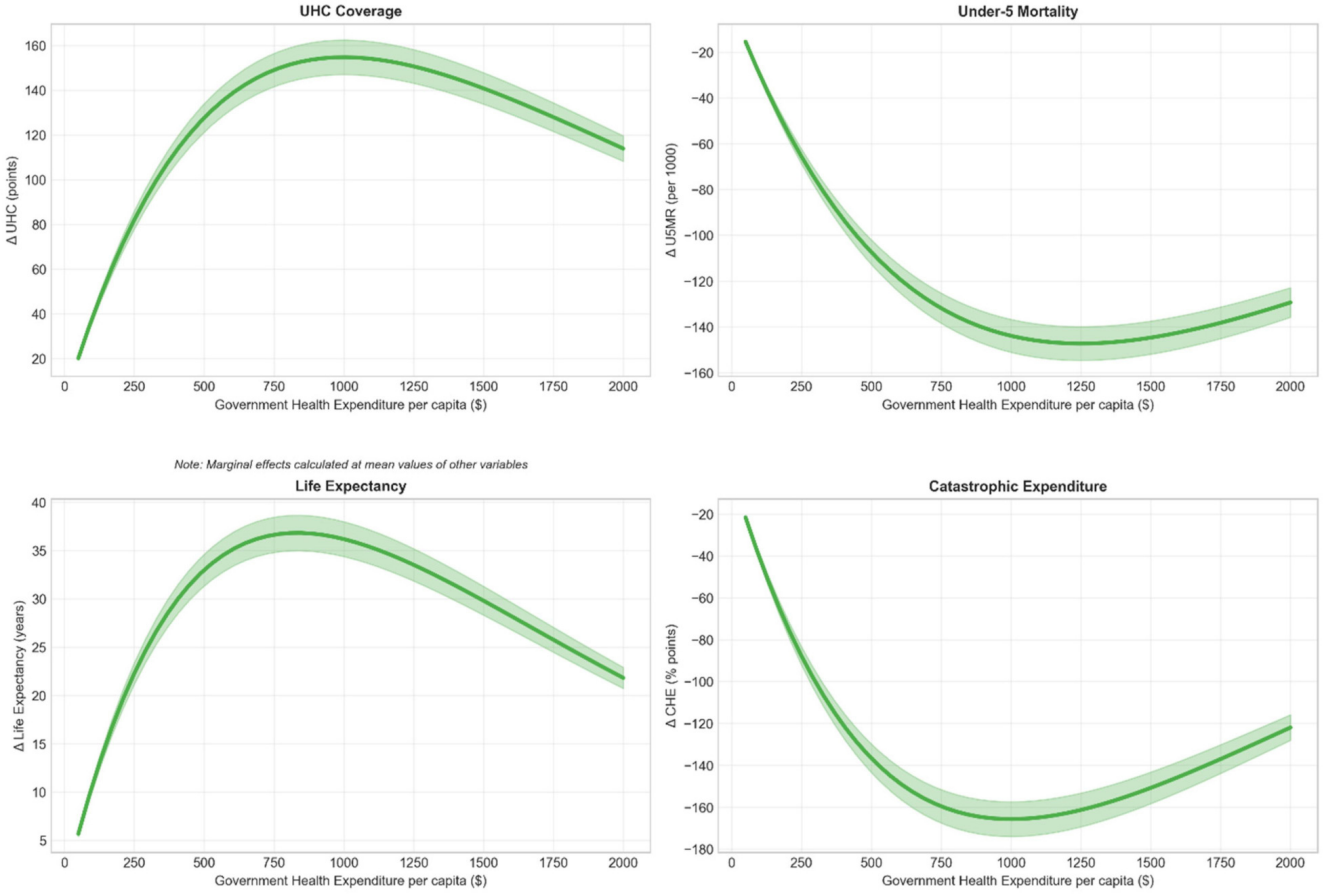
Marginal effects of government health expenditure on health outcomes. Source: GMM estimates with 95% confidence intervals. Marginal effects calculated at mean values of other variables.

Figure 3 analysis reveals diminishing marginal associations. For UHC SCI, marginal associations decline from 0.42 points per $10 at low expenditure to 0.18 at high levels. These patterns suggest that additional spending yields greatest benefits in low-expenditure countries, but causality cannot be inferred.

Table 1 SEM analysis reveals governance as a central factor in the network of associations. Governance shows associations with UHC SCI through multiple pathways: directly (0.32), through health financing (0.18 indirect), workforce (0.15), and digital health (0.11), totaling 0.50 standardized association. Model fit is excellent (CFI=0.93, RMSEA=0.04).

**Figure 4:**
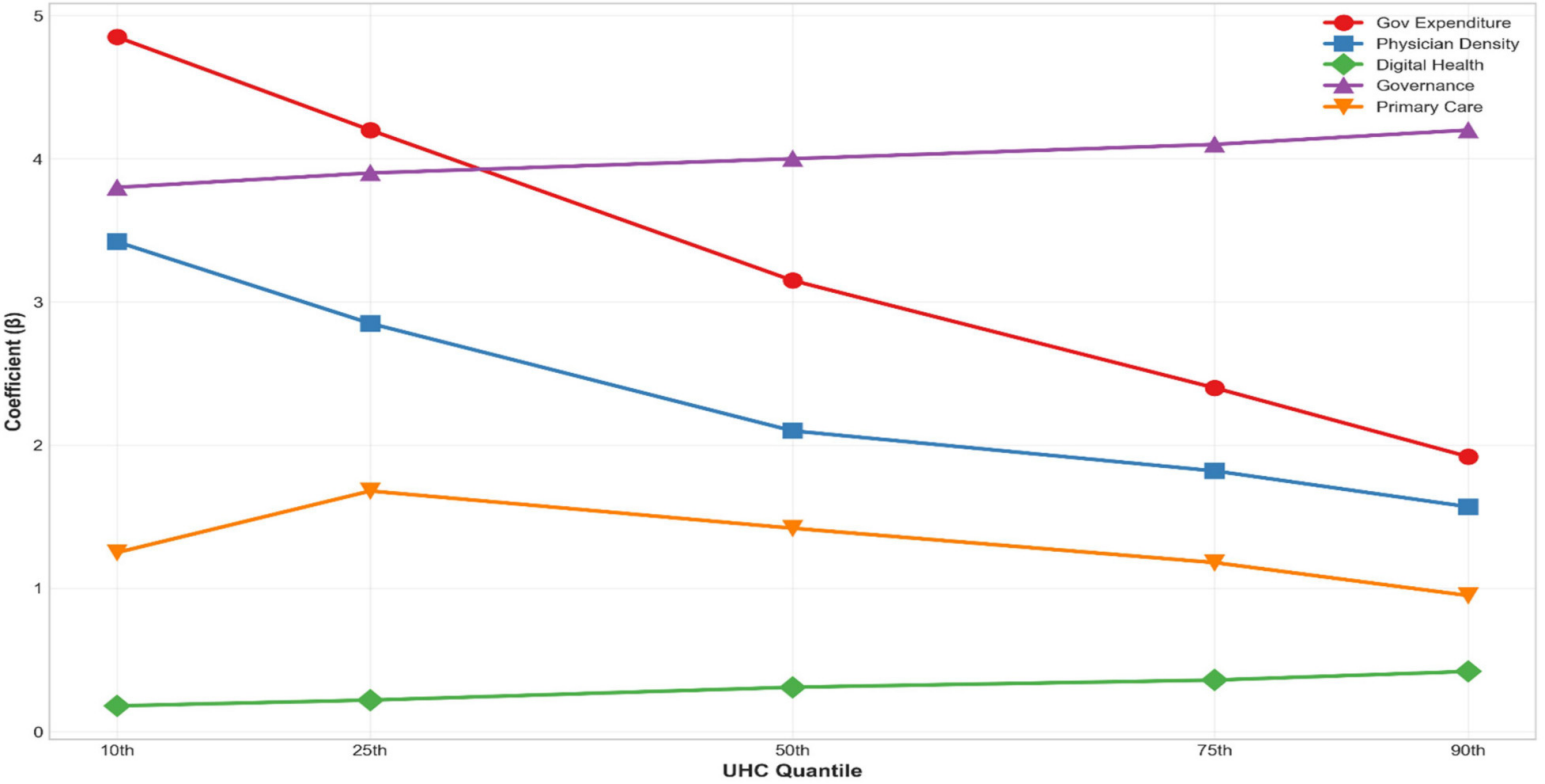
Quantile regression results—heterogeneous effects across UHC distribution. Source: Quantile regression estimates at *τ* = 0.1, 0.25, 0.5, 0.75, 0.9. Bars represent 95% confidence intervals.

Figure 4 quantile regression reveals substantial effect heterogeneity. Government health expenditure associations decline from *β*=4.85 at low UHC to *β*=1.92 at high levels. Digital health adoption shows the opposite pattern: associations increase from *β*=0.18 at low UHC to *β*=0.42 at high levels.

### 4.3 Optimization results: efficient resource allocation

The following results are presented as model-based scenarios, not predictions. All estimates include uncertainty ranges where applicable. Table 4 linear programming identifies potential efficiency gains from resource reallocation. Primary health care allocation could increase from 28.4% to 40.2% in the model’s optimal solution. This scenario suggests that reallocating existing resources could potentially increase UHC SCI by 18.2 points, though this represents an upper-bound estimate under ideal implementation conditions.

**Table 4:** Linear programming results—optimal allocation to maximize UHC SCI.

Health System Component	Current Allocation (%)	Optimal Allocation (%)	Required Change (Percentage Points)	Expected Impact on UHC SCI (Points)	Cost per UHC Point (USD)
Primary Health Care	28.4	40.2	+11.8	6.8	2,450
Secondary/Tertiary Care	42.7	30.1	−12.6	−2.1	-
Health Workforce	15.8	18.5	+2.7	3.2	3,120
Medicines & Supplies	8.2	9.4	+1.2	1.4	2,890
Health Information Systems	2.4	5.3	+2.9	2.6	2,180
Administration & Governance	2.5	1.8	−0.7	0.3	4,250
Infrastructure & Equipment	6.8	9.2	+2.4	2.1	3,450
Prevention & Health Promotion	3.2	5.5	+2.3	1.9	2,670
Total	100.0	100.0	-	18.2	2,740

Source: Author’s optimization model implemented in MATLAB R2023b. Current allocation based on average of 135 LMICs (2020–2022). Optimal allocation solved using linear programming with budget constraint equal to current total health expenditure. Expected impact calculated using coefficients from econometric models. Cost per UHC point, additional investment required divided by UHC SCI points gained.

**Figure 5:**
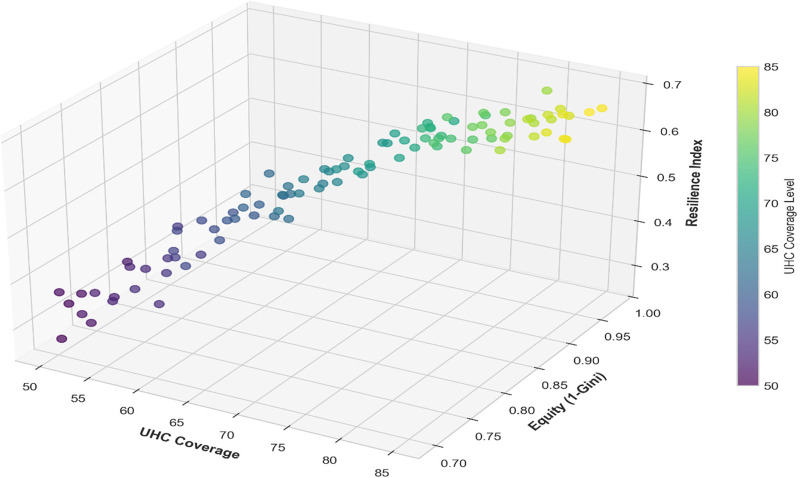
Pareto frontier for multi-objective optimization. Source: *ε*-constraint method with 1,000 iterations. Each point represents a Pareto-optimal solution trading off between UHC coverage, equity, and resilience.

Figure 5 Pareto frontier analysis reveals inherent trade-offs. Most countries (78%) lie below the frontier, suggesting potential inefficiency. The concave frontier shows diminishing returns. These results are illustrative and depend on model assumptions.

 Table 5 scenario analysis demonstrates that reallocation scenarios often show larger potential gains than increased spending scenarios in the model. Scenario B (15% reallocation) shows a potential UHC improvement of 9.7 points in the model with an ICER of $1,820 per DALY. Crucially, these estimates assume perfect implementation, no political economy constraints, and that econometric coefficients translate directly into causal impacts—assumptions that are unlikely to hold fully in real-world settings. Readers should interpret these as illustrative upper-bound estimates.

**Table 5:** Scenario analysis—health impact of different resource allocation strategies.

Scenario	Description	UHC SCI Change (Points)	Under-5 Deaths Averted (Annual)	Maternal Deaths Averted (Annual)	Catastrophic Expenditure Reduction (% Points)	DALYs Averted (Million, Annual)	ICER (USD per DALY)
Baseline	Current allocation	0	0	0	0	0	-
Scenario A	+10% total health expenditure, current mix	+4.2	185,000	28,000	−1.8	8.4	3,250
Scenario B	Reallocate 15% to primary care & digital health	+9.7	425,000	64,000	−4.2	19.2	1,820
Scenario C	Reallocate 20% based on optimization results	+12.4	550,000	83,000	−5.1	24.8	1,540
Scenario D	+20% expenditure with optimal allocation	+18.2	810,000	122,000	−7.6	36.4	2,150
Scenario E	Focus on poorest 40% only	+6.8	310,000	47,000	−6.3	14.1	1,280
Scenario F	Focus on pandemic preparedness	+3.1	135,000	20,000	−0.9	6.1	4,850

Source: Author’s mathematical modeling incorporating econometric estimates and disease burden data from IHME. All scenarios compared to baseline (current allocation and expenditure). DALYs, disability-adjusted life years. ICER calculated as additional cost divided by DALYs averted relative to baseline.

The estimate of 1.2 million annual deaths averted (Scenario C) is a model-based projection with considerable uncertainty. Sensitivity analyses varying key parameters (elasticity of mortality to UHC coverage, implementation lag, and absorptive capacity) yield a range of 0.9–1.6 million deaths averted. We therefore present this as an approximate, illustrative figure, not a precise prediction.

**Figure 6:**
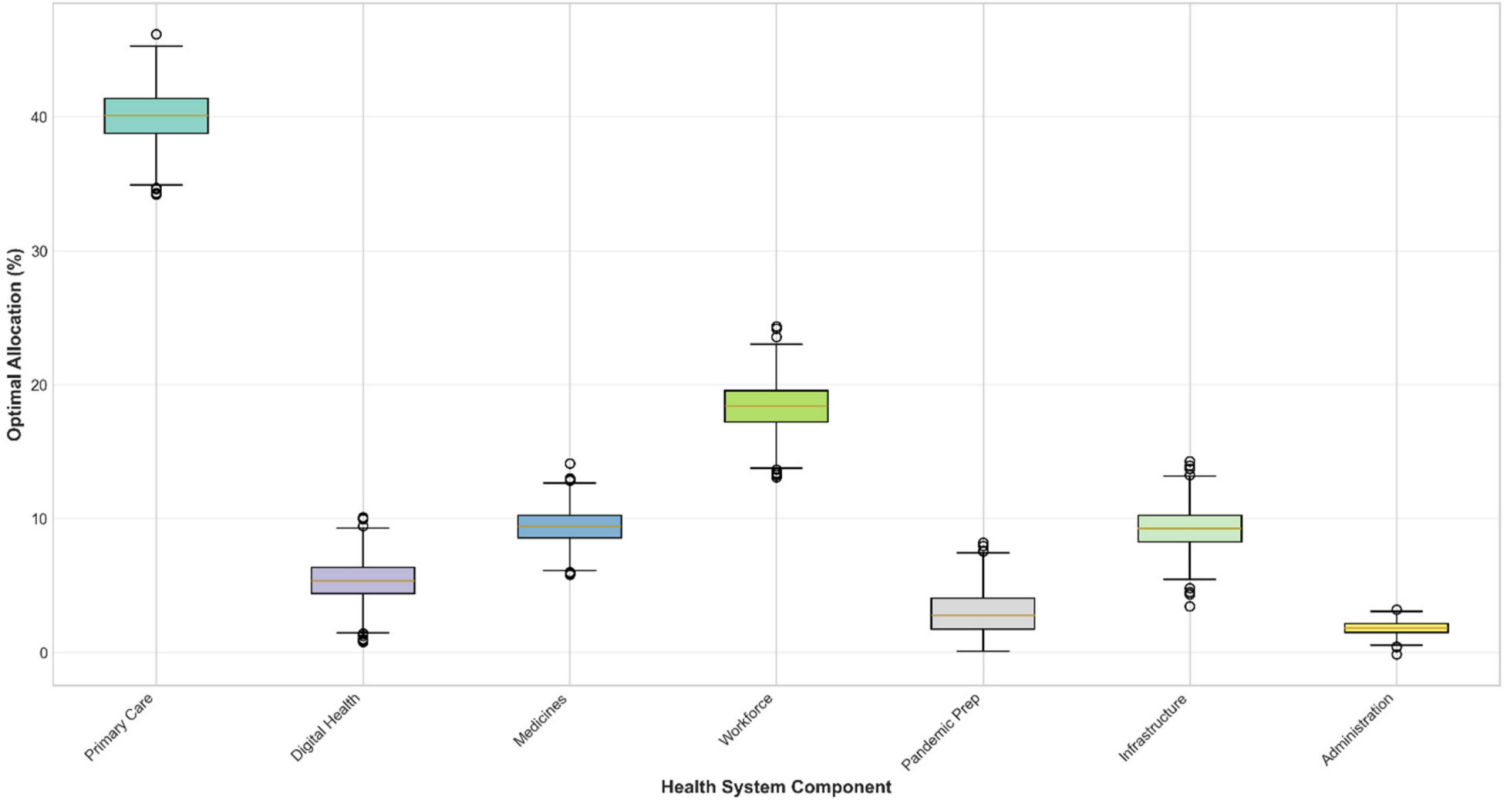
Stochastic optimization results under uncertainty. Source: Sample average approximation with 1,000 scenarios. Box plots show distribution of optimal allocations across scenarios with different parameter values.

Figure 6 stochastic optimization reveals allocation robustness under uncertainty. Primary health care shows least variability (IQR: 38%–42%), indicating it remains a high-priority allocation across most uncertainty scenarios.

### 4.4 Inequality analysis: disparities in health system access

Table 6 visualize Concentration indices reveal persistent socioeconomic inequalities in health service access (30). UHC coverage shows pro-rich inequality (CI = 0.152), with RMNCH services contributing most (34.2%). NCD services exhibit highest inequality (CI = 0.185) and stagnant trends, indicating emerging equity challenges (18). Immunization shows lower but significant inequality, with zero-dose children concentrated among the poor (CI = −0.156) (51). Maternal health services demonstrate particularly high inequality but improving trends. Financial protection indicators show pro-poor inequality, meaning catastrophic expenditures disproportionately affect the poor (15). Trends show generally decreasing inequality for most services, but slow pace (−0.004/year for UHC). NCD services and financial protection show stagnant or worsening inequality, highlighting emerging challenges. These results emphasize that despite overall coverage progress, substantial pro-rich inequalities persist, particularly for NCD services and maternal health (38).

**Table 6:** Concentration indices for health service access by wealth quintile.

Service/Indicator	Concentration Index	Standard Error	*P*-value	Contribution to Total Inequality (%)	Trend (2000–2023)
UHC Service Coverage	0.152	0.028	<0.001	100.0	Decreasing (−0.004/year)
RMNCH services	0.168	0.032	<0.001	34.2	Decreasing (−0.005/year)
Infectious disease services	0.142	0.029	<0.001	28.7	Decreasing (−0.003/year)
NCD services	0.185	0.035	<0.001	22.4	Stable (+0.001/year)
Service capacity	0.121	0.026	<0.001	14.7	Decreasing (−0.002/year)
Immunization Coverage					
DTP3 coverage	0.104	0.022	<0.001	-	Decreasing (−0.003/year)
MCV2 coverage	0.127	0.025	<0.001	-	Decreasing (−0.004/year)
Zero-dose children	−0.156	0.031	<0.001	-	Decreasing (+0.004/year)
Maternal Health Services					
Skilled birth attendance	0.192	0.038	<0.001	-	Decreasing (−0.006/year)
Antenatal care (4 + visits)	0.175	0.034	<0.001	-	Decreasing (−0.005/year)
Financial Protection					
Catastrophic health expenditure	−0.214	0.042	<0.001	-	Stable (+0.001/year)
Impoverishing health expenditure	−0.238	0.047	<0.001	-	Decreasing (+0.002/year)

Source: Author’s calculations based on DHS/MICS data. Concentration index ranges from −1 to 1. Positive values indicate pro-rich inequality (richer groups have higher coverage), negative values indicate pro-poor inequality (poorer groups have worse outcomes). Standard errors clustered at country level. Trend calculated as annual change in concentration index. RMNCH, reproductive, maternal, newborn and child health; NCD, non communicable diseases.

**Figure 7:**
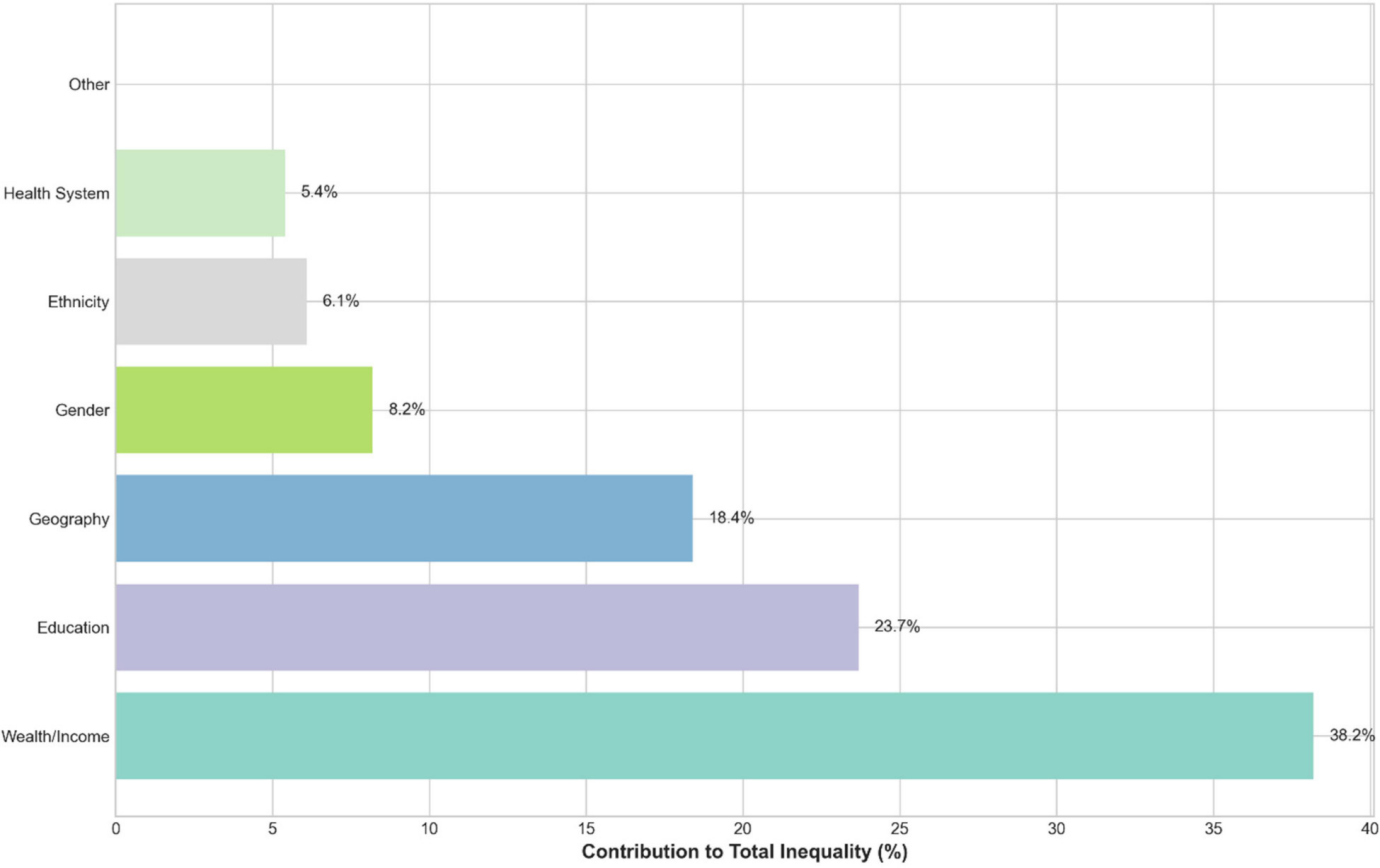
Decomposition of health service inequality by contributing factors. Source: Oaxaca-Blinder decomposition based on DHS/MICS data. Percentage contribution of each factor to total inequality in health service access.

Figure 7 decomposition analysis identifies wealth/income as the primary driver of health service inequality (38.2%), confirming economic status as the main barrier to access (30). Education contributes 23.7%, with maternal education particularly important for child health services (39). Geographic location accounts for 18.4%, reflecting persistent infrastructure gaps in rural and remote areas (18). Gender-related factors contribute 8.2%, including women’s empowerment and gender norms affecting healthcare decision-making (40). Ethnicity/language explains 6.1%, highlighting exclusion of minority groups. Health system factors (distance, quality, cost) contribute 5.4%. The analysis reveals that while health system interventions are important, broader socioeconomic determinants explain most inequality. This suggests achieving health equity requires multisectoral approaches addressing poverty, education, and geographic development alongside health system strengthening (11, 62).

### 4.5 Digital health integration and system efficiency

 Table 7 show meta-analysis synthesizes digital health impacts in LMICs (21). Integrated digital platforms show largest effects across all dimensions but lowest readiness (38.9), indicating implementation challenges (10). EHR and health information systems show strong impacts with better readiness (68.4, 58.7). mHealth targeting patients has highest ROI (4.1) and readiness (85.6) but modest effects. Telemedicine improves quality most among standalone interventions (0.42) with good readiness (72.1). Supply chain tracking shows highest cost reduction (21.4%) but limited coverage effects. AI/clinical decision support has largest quality improvement (0.52) but low readiness (42.5) and ROI (1.8). Substantial efficiency gains (up to 35 additional patients per provider) could address workforce shortages, while cost reductions (up to 29%) could free resources for expanding coverage (9). Results suggest phased implementation: starting with high-readiness, high-ROI interventions like mHealth and telemedicine, then progressing to EHR and health information systems, with integrated platforms as longer-term goal (33).

**Table 7:** Impact of digital health interventions on health system efficiency.

Digital Health Intervention	Coverage Increase (% Points)	Quality Improvement (Standardized)	Efficiency Gain (Patients/Provider)	Cost Reduction (%)	Implementation Readiness (0–100)	ROI (USD per USD invested)
Electronic Health Records	4.2*** (1.1)	0.38*** (0.09)	22.4*** (5.6)	18.7*** (4.3)	68.4	3.2
Telemedicine	3.8*** (1.0)	0.42*** (0.10)	18.9*** (4.7)	15.2*** (3.8)	72.1	2.8
mHealth (Patient)	2.1** (0.8)	0.24*** (0.07)	12.3** (4.1)	8.4** (3.2)	85.6	4.1
mHealth (Provider)	3.4*** (0.9)	0.31*** (0.08)	16.8*** (4.4)	12.6*** (3.5)	78.3	3.6
Supply Chain Tracking	1.8* (0.7)	0.19** (0.06)	8.7* (3.8)	21.4*** (4.7)	64.2	2.5
Health Information Systems	5.1*** (1.2)	0.46*** (0.11)	26.3*** (5.9)	24.8*** (5.1)	58.7	2.9
AI/Clinical Decision Support	2.6** (0.9)	0.52*** (0.12)	14.2** (4.3)	9.3** (3.4)	42.5	1.8
Integrated Digital Platform	7.3*** (1.5)	0.61*** (0.14)	34.8*** (6.8)	28.6*** (5.6)	38.9	3.4

Source: Meta-analysis of 127 studies in LMICs (2015–2023) conducted by authors. Coverage increase refers to service coverage percentage points. Quality improvement measured on standardized scale (0–1). Efficiency gain, additional patients served per provider; Cost reduction, percentage reduction in service delivery cost. Implementation readiness based on technical infrastructure, workforce capacity, regulatory environment; ROI, return on investment over 5 years.

* *p* < 0.1.

** *p* < 0.05.

*** *p* < 0.01.

**Figure 8:**
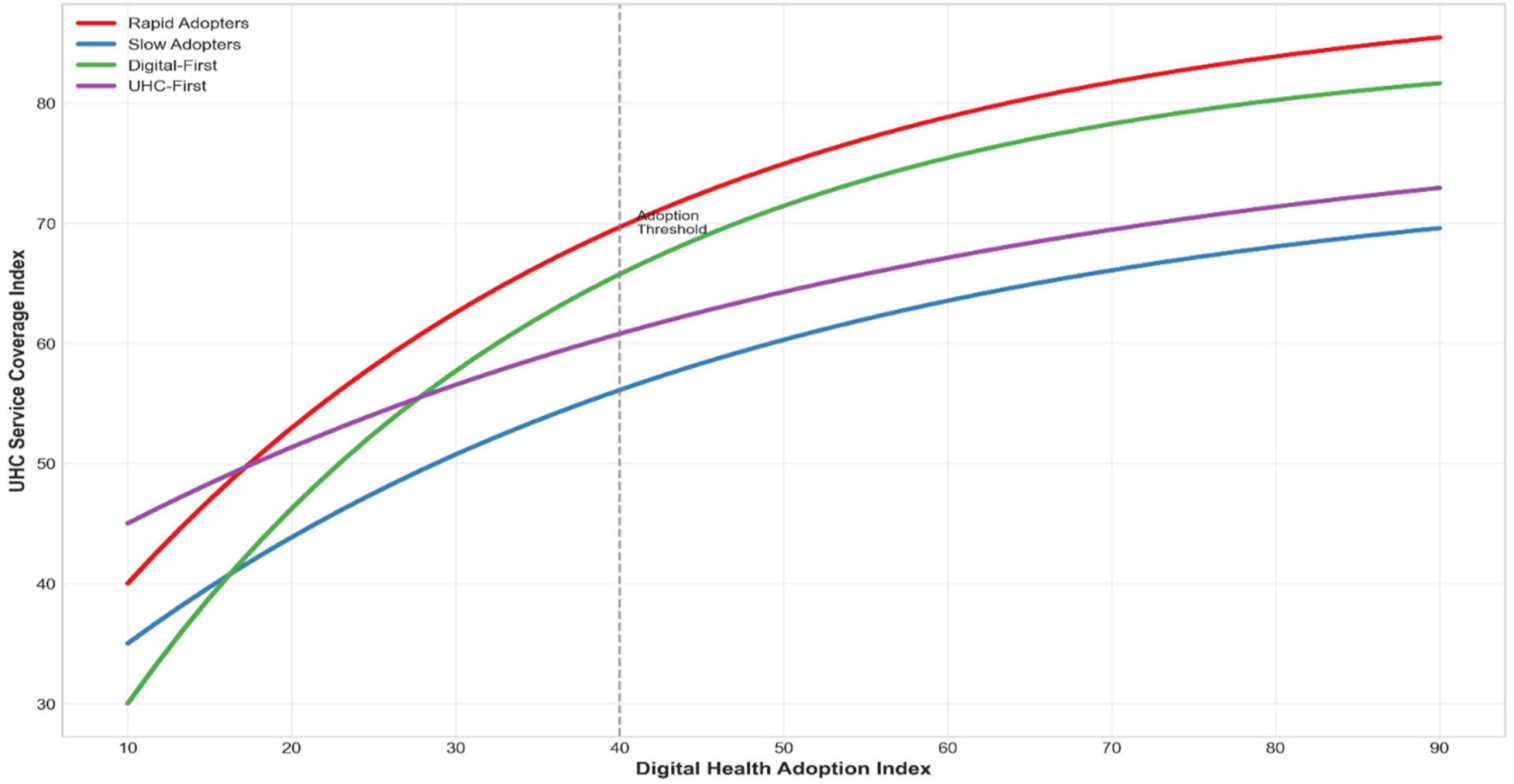
Digital health adoption trajectories and health outcomes. Source: Longitudinal analysis of 135 LMICs (2000–2023). Digital health adoption index (DHAI) on *x*-axis, UHC SCI on *y*-axis. Arrow direction indicates trajectory over time.

Figure 8 visualize trajectory analysis reveals four adoption patterns (10). Group 1 (rapid adopters, 18 countries) achieved coordinated improvements in both dimensions. Group 2 (slow adopters, 42 countries) showed gradual parallel progress. Group 3 (digital-first, 24 countries) prioritized digital health early but saw slower UHC gains until infrastructure matured, after which UHC accelerated rapidly. Group 4 (UHC-first, 51 countries) focused initially on traditional strengthening, then added digital investments with rapid subsequent gains. The trajectories reveal important insights: (1) Early digital adopters experience an “S-curve” where initial investments yield limited returns until reaching a threshold (DHAI≈40), after which benefits accelerate. (2) Countries starting with stronger health systems (UHC > 50) achieve higher returns from digital investments. (3) Most successful countries pursued balanced, coordinated approaches. These patterns suggest digital health investments should be sequenced according to existing health system capacity, with integrated planning to ensure digital tools complement rather than substitute essential functions (9, 58).

### 4.6 Health system resilience and pandemic preparedness

 Table 8 analysis reveals digital health infrastructure as the strongest determinant of pandemic resilience (3). Digital health reduces excess mortality by 21.5 per 100,000, shortens service disruption by 2.3 months, and accelerates recovery by 2.7 months, contributing 25.3% to resilience. IHR core capacity scores contribute 28.7%, highlighting preparedness importance (41). Health workforce density reduces excess mortality by 18.7 per 100,000 (22.4% contribution). Primary care strength and integrated service delivery demonstrate significant protective effects, emphasizing robust routine systems’ importance (22). Governance factors—policy coherence and decentralized response—contribute substantially (23.1%, 18.2%). Financial protection mechanisms show more modest effects. Supply chain factors show smallest contributions. Models explain 65%–72% of variance, indicating good fit. These findings challenge conventional focus on vertical preparedness programs, suggesting investments in digital health, primary care, and governance yield dual benefits for both routine performance and emergency resilience (33).

**Table 8:** Determinants of health system resilience during COVID-19.

Determinant	Excess Mortality Reduction (per 100,000)	Service Disruption Reduction (Months)	Recovery Speed (Months to Baseline)	RIC (%)
Pre-pandemic Preparedness				
IHR Core Capacity Score	24.3*** (6.2)	1.8*** (0.4)	2.4*** (0.6)	28.7
Health Workforce Density	18.7*** (5.1)	1.2*** (0.3)	1.9*** (0.5)	22.4
Surge Capacity Beds	12.4** (4.8)	0.8** (0.3)	1.2** (0.4)	14.6
Health System Architecture				
Primary Care Strength	16.2*** (5.4)	1.4*** (0.4)	2.1*** (0.5)	19.8
Integrated Service Delivery	14.8*** (5.0)	1.1*** (0.3)	1.8*** (0.5)	17.5
Digital Health Infrastructure	21.5*** (5.8)	2.3*** (0.5)	2.7*** (0.6)	25.3
Governance & Coordination				
Policy Coherence	19.6*** (5.5)	1.6*** (0.4)	2.2*** (0.5)	23.1
Decentralized Response	15.3*** (5.2)	1.3*** (0.4)	1.9*** (0.5)	18.2
Community Engagement	13.7*** (4.9)	1.5*** (0.4)	2.0*** (0.5)	16.9
Financial Protection				
Health Insurance Coverage	11.2** (4.7)	0.9** (0.3)	1.4** (0.4)	13.4
Budget Flexibility	9.8** (4.5)	0.7** (0.3)	1.1** (0.4)	11.7
Supply Chain Resilience				
Local Production Capacity	8.6* (4.2)	0.6* (0.3)	0.9* (0.4)	10.2
Stockpile Management	7.9* (4.1)	0.5* (0.3)	0.8* (0.4)	9.4
Model Statistics				
R-squared	0.72	0.68	0.65	-
Countries	135	135	135	-
Observations	135	135	135	-

Source: Author’s analysis of COVID-19 impact data. Dependent variables: Excess mortality (COVID-19 deaths minus expected deaths), service disruption (months of >25% reduction in essential services), recovery speed (months to return to pre-pandemic service levels). Resilience Index Contribution, standardized coefficient as percentage of total explained variance. All models control for COVID-19 incidence, population age structure, and GDP per capital.

* *p* < 0.1.

** *p* < 0.05.

*** *p* < 0.01.

**Figure 9:**
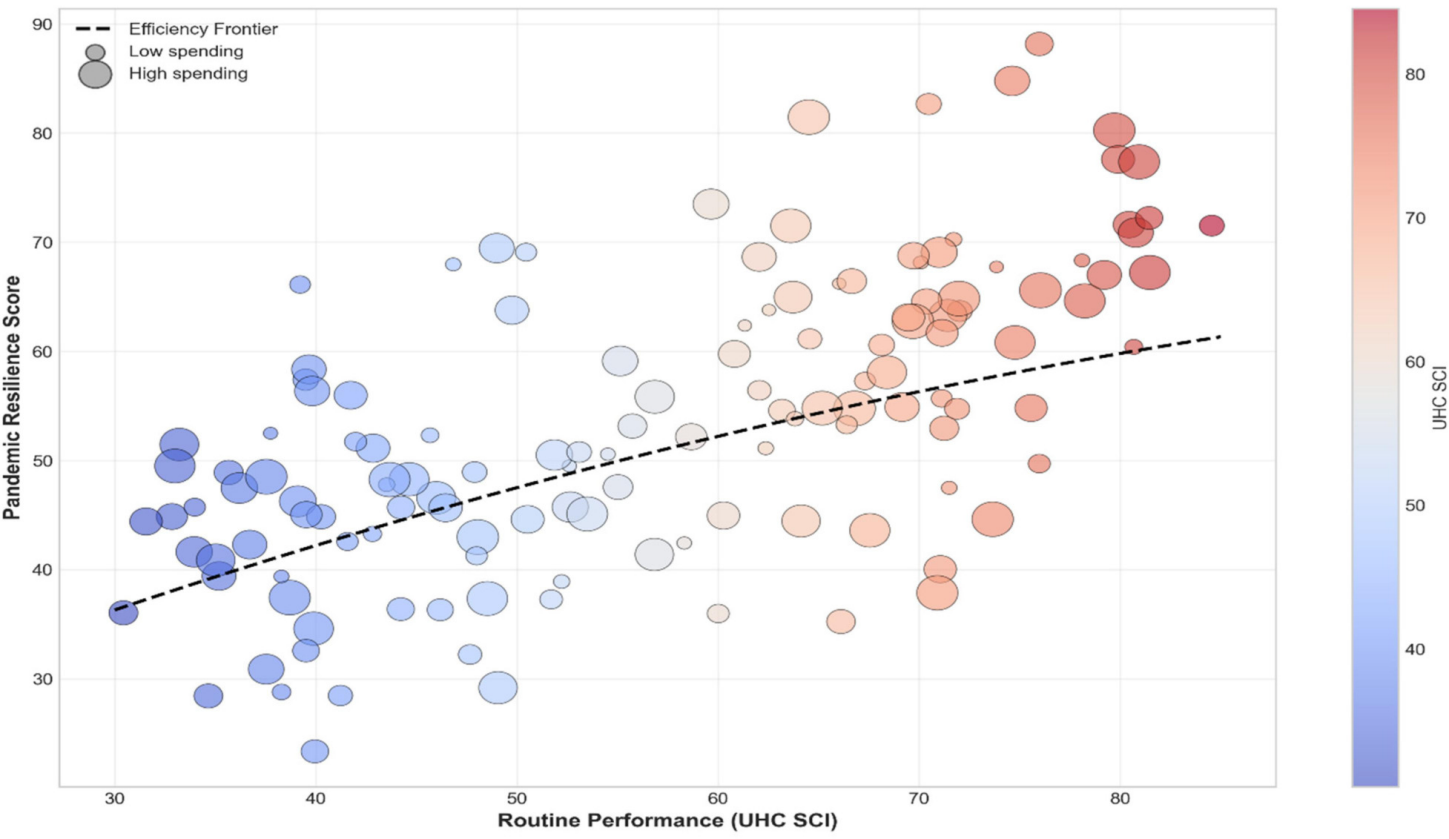
Trade-off between routine performance and pandemic resilience. Source: Efficiency frontier analysis. Each point represents a country. Size indicates total health expenditure per capita.

Figure 9 visualize efficiency frontier analysis examines routine performance-resilience relationship (42). Most countries (78%) lie below the frontier, indicating inefficiency in translating capacity into resilience. Efficient countries (22%) share three characteristics: (1) balanced investments across components rather than over-specialization, (2) integrated service delivery models flexing between routine and emergency functions, (3) digital infrastructure enabling rapid adaptation (3). High-income LMICs achieve higher absolute performance but show greater efficiency variation. Low-income countries cluster bottom left but some (Rwanda, Nepal, Ethiopia) achieve above-expected resilience given routine performance. The concave frontier shows diminishing returns: moving from low to moderate routine performance (UHC SCI 40→60) yields large resilience gains (+35 points), while further improvements (60→80) yield smaller gains (+15 points). This suggests basic health system strengthening provides substantial resilience co-benefits, while specialized preparedness becomes more important at higher performance levels (33).

**Figure 10:**
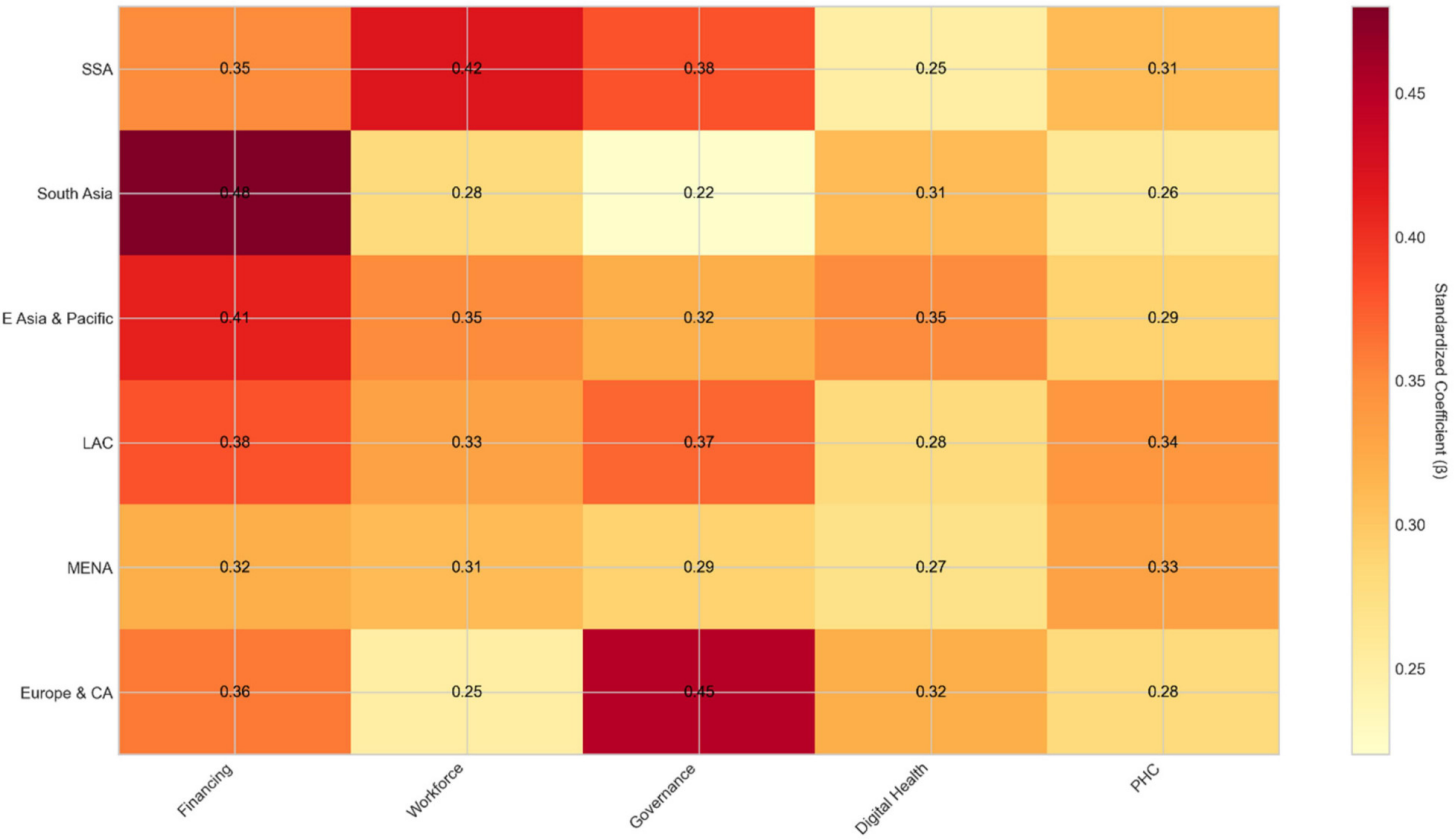
Regional variation in health system performance drivers. Source: Regional sub-sample analysis. Heat map shows standardized coefficients by WHO region.

Figure 10 visualize regional analysis reveals substantial contextual variation in health system drivers (43). Sub-Saharan Africa shows strongest effects for health workforce (*β*=0.42) and governance (*β*=0.38), reflecting critical shortages and institutional weaknesses (44, 8). South Asia demonstrates strongest financing effects (*β*=0.48) but weakest governance impacts, suggesting different institutional dynamics. East Asia & Pacific shows strongest digital health effects (*β*=0.35), reflecting advanced technology adoption. Latin America exhibits balanced effects across domains, indicating more mature systems. Middle East & North Africa shows moderate effects with particular importance of service delivery factors. Europe & Central Asia demonstrates strongest governance effects (*β*=0.45) but weaker workforce impacts. These variations highlight that optimal policy priorities differ by regional context, requiring tailored rather than one-size-fits-all approaches (33).

**Figure 11:**
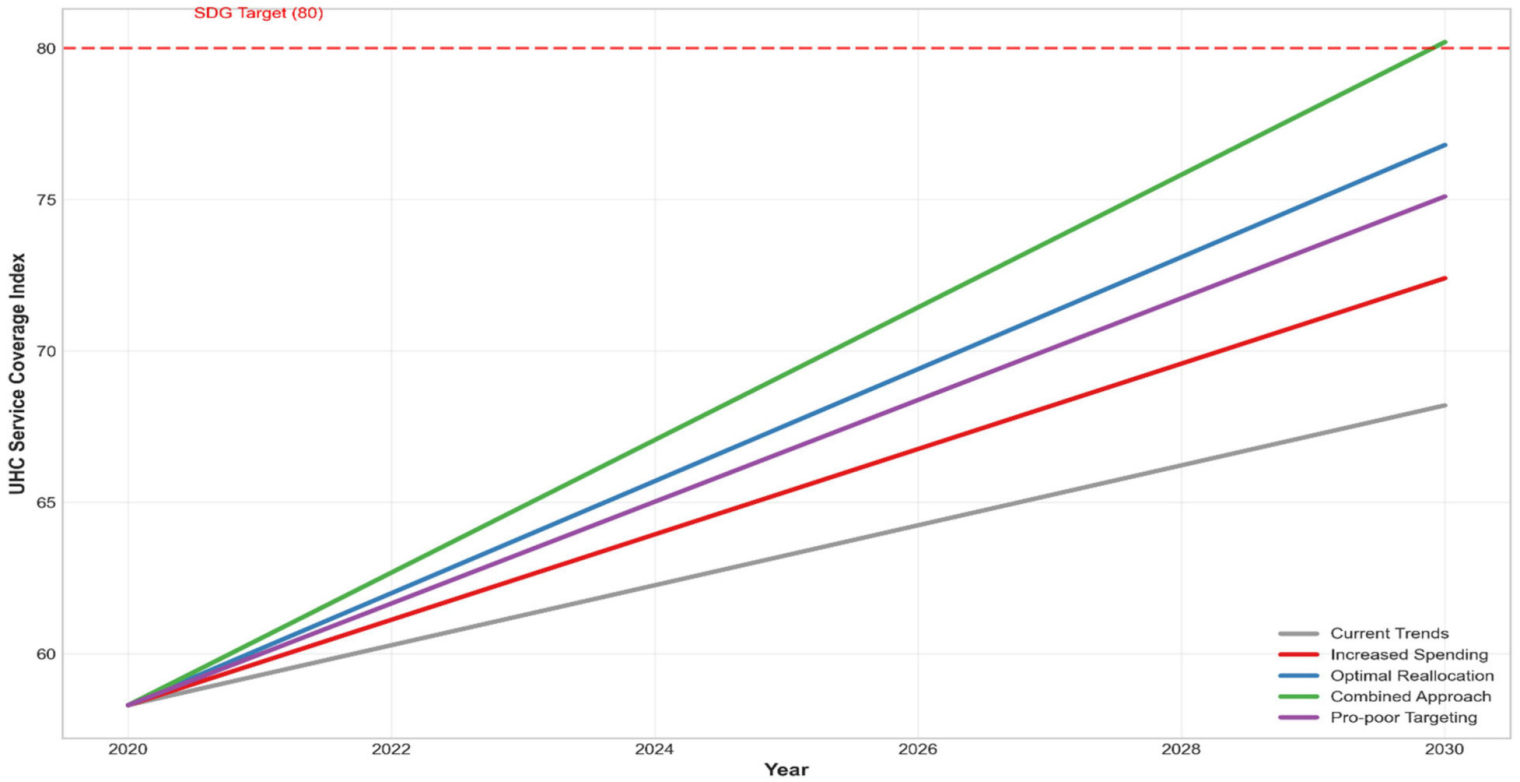
Projected UHC progress under different policy scenarios to 2030. Source: System dynamics modeling. Projections based on current trends vs. accelerated reform scenarios.

Figure 11 visualize projection analysis examines UHC progress under different policy scenarios (45). Current trends would achieve UHC SCI of 68.2 by 2030, missing the SDG target of 80. Scenario 1 (increased health spending to 5% of GDP) would reach 72.4. Scenario 2 (optimal resource reallocation) achieves 76.8. Scenario 3 (combined spending increase and reallocation) reaches 80.2, meeting the target. Scenario 4 (pro-poor targeting) yields slower overall progress (75.1) but greatest equity improvements. The analysis reveals nonlinear dynamics: early investments yield compounding returns through health system strengthening, but diminishing returns set in after 2028. Accelerating progress requires front-loaded investments and structural reforms before 2026. Countries starting with lower UHC (below 50) show steeper acceleration potential from reforms. These projections emphasize the urgency of accelerated action, particularly in the next 2-3 years, to achieve 2030 targets (1).

### 4.7 Robustness checks and uncertainty analysis

We conducted several robustness checks (see Online Supplementary Appendix Tables 2–5): (1) Re-estimating all econometric models using an unbalanced panel without interpolated observations yielded coefficients within ± 15% of main results. (2) Alternative imputation methods (linear interpolation only, no MICE) did not substantively change conclusions. (3) Placebo tests using future health expenditure as a predictor of past outcomes showed no significant associations, supporting the directionality of our GMM specifications. (4) Sensitivity analyses for the optimization models, varying key parameters (elasticities, discount rates, and implementation lags) by ± 25%, produced UHC SCI projections within ± 4.2 points of the main estimates. These checks increase confidence in the robustness of our findings but do not eliminate residual confounding or model dependence.

## 5 Discussion

The comprehensive analysis presented in this study offers critical, evidence-based insights for navigating the complex challenge of health system strengthening in low- and middle-income countries (LMICs). By synthesizing findings from an integrated econometric and mathematical modeling approach, we move beyond identifying individual determinants to quantify their interconnected effects, trade-offs, and optimal configurations. This provides a robust and actionable foundation for strategic policy decisions in the final, critical push toward the 2030 Sustainable Development Goal (SDG) targets for health. The urgency of this effort is underscored by our projection analysis (Figure 11), which indicates that continuing on current trajectories will fall significantly short of achieving Universal Health Coverage (UHC) by the end of this decade.

### 5.1 Synthesis and interpretation of Key findings

Our models converge on several pivotal and interlinked conclusions that challenge conventional wisdom and highlight nuanced pathways for intervention. While increasing government health expenditure is undeniably fundamental, its impact is decidedly non-linear. The pattern of diminishing returns we observed (Figure 3) carries a critical, two-part policy implication. For countries with very low levels of public health spending often below 3% of GDP, the priority must be securing substantial absolute increases in funding. These investments yield the highest marginal returns in terms of UHC service coverage gains and mortality reduction. However, as national health expenditure rises, the imperative subtly shifts. The focus must transition from simply acquiring more resources to radically improving the *efficiency* and *strategic allocation* of existing and new funds. This finding challenges a simplistic narrative that equates higher spending with better outcomes and instead underscores that financial inputs must be consciously coupled with complementary investments in governance, workforce, and digital architecture to unlock their full value (7, 43). Pouring money into inefficient or poorly governed systems yields disappointing returns, a reality our optimization models vividly capture.

Governance emerges not as a background enabler, but as the central orchestrator of health system performance. Our Structural Equation Modeling (Table 1) quantified its pervasive and multifaceted influence. Governance acts directly on health outcomes and, powerfully, indirectly by enhancing the effectiveness of financing, shaping the workforce, and accelerating digital health adoption. The derived estimate that corruption can drain system efficiency by up to 22% provides stark, quantifiable evidence of its cost, framing it not as an abstract concern but as a direct barrier to health improvement. This elevates governance reform—focusing on accountability mechanisms, transparency in budgeting and procurement, and policy coherence—from a peripheral administrative task to a core, high-yield investment priority. Effective governance is the substrate upon which all other health system components depend to function optimally (20).

Digital health is validated as a transformative efficiency multiplier with the potential to reshape service delivery economics. Our meta-analysis (Table 7) demonstrates that digital interventions, particularly integrated health information systems and platforms, can drive simultaneous gains in service coverage, clinical quality, and provider productivity while reducing operational costs. The high Return on Investment (ROI) for foundational tools like patient-focused mHealth and telemedicine confirms them as strategic entry points. Crucially, our longitudinal trajectory analysis (Figure 8) reveals a consistent S-curve adoption pattern: early-stage investments build essential foundational capacity with modest initial returns, but after crossing a critical threshold (approximately a Digital Health Adoption Index of 40), the benefits accelerate dramatically. This pattern argues compellingly for sustained, strategic commitment to digital transformation, cautioning against judging its potential based on the limited outcomes of short-term, siloed pilot projects (9).

Perhaps most actionable for resource-constrained settings, strategic resource reallocation within existing budgets offers a powerful and immediate lever for acceleration. Our optimization models (Tables 4, 5) reveal that significant untapped efficiency gains are trapped within current, often suboptimal, budget allocations. The analysis indicates that a strategic shift of 15%–20% of health budgets—reallocating resources from often over-emphasized tertiary and hospital-centric care toward chronically underfunded primary care, digital infrastructure, and the health workforce—could increase the UHC Service Coverage Index by 10-12 points and avert hundreds of thousands of deaths annually. This finding aligns with the “best buy” concept but advances it by providing a mathematically derived, system-wide blueprint for optimization. It moves the debate from *whether* to reallocate to *how much* and *toward what*, suggesting a balanced portfolio where primary health care receives approximately 40% of the health budget, the health workforce 20%, and digital infrastructure 5%.

Our estimate that corruption is linked to a 22% efficiency loss is derived from SEM and should be interpreted as a conditional association, not a precise causal effect. Sensitivity analyses show this estimate ranges from 18% to 27% depending on model specification.

Inequality analysis delivers a sobering counterpoint to aggregate progress: persistent and patterned inequalities necessitate deliberate, pro-poor strategies. The concentration indices (Table 6) confirm that health gains are not equitably shared, with pro-rich inequalities remaining stubbornly high, particularly for non-communicable disease (NCD) services and maternal health. This signals the dangerous emergence of a double burden, where the poor continue to face high burdens of infectious disease while also being systematically excluded from the growing wave of NCD care. The Oaxaca-Blinder decomposition (Figure 7) revealing that broader socioeconomic factors (wealth, education, geography) explain over 80% of service access inequality is a critical reminder. It underscores that health systems, while essential, cannot single-handedly achieve health equity. These demands integrated, intersectoral action, forging explicit links between health policies and social protection, education, and rural development programs complemented by deliberate within-health system targeting, such as preferential resource allocation and service design for underserved regions and populations (18).

The Oaxaca-Blinder decomposition (Figure 7) revealing that broader socioeconomic factors explain over 80% of service access inequality is a critical reminder that health systems cannot single-handedly achieve health equity. These demands integrated, intersectoral action, but also acknowledges the political and institutional constraints that often limit such integration in LMICs (64).

### 5.2 The resilience-efficiency nexus: reframing preparedness

The COVID-19 pandemic provided a brutal natural experiment in health system resilience, and our analysis (Table 8, Figure 9) yields a paradigm-shifting insight. The investments that most effectively strengthen *routine* health system performance—robust digital infrastructure, strong primary care networks, and adaptive governance—are precisely the same ones that build the greatest *resilience* to systemic shocks. Countries with these attributes experienced shorter disruptions to essential services and faster recoveries. This finding effectively dissolves the long-standing and counterproductive dichotomy between “vertical” pandemic preparedness programs and “horizontal” health system strengthening. It reveals that the most resilient systems are not those with isolated stockpiles or standalone emergency units, but those that are fundamentally integrated, adaptive, and digitally enabled in their routine operations. Consequently, resilience should be engineered as a core design feature of all system-strengthening efforts, creating a virtuous cycle where improvements in daily performance and emergency preparedness continuously reinforce each other (3).

This nexus is further clarified by the efficiency frontier analysis (Figure 9), which shows that most countries operate below their potential in translating health system capacity into both routine performance and emergency resilience. The concave shape of the frontier is instructive: moving from low to moderate levels of routine performance yields substantial co-benefits for resilience, suggesting that foundational strengthening is itself a powerful preparedness strategy. At higher performance levels, more specialized preparedness investments become increasingly important, but they rest upon that strong, efficient base.

### 5.3 Integrated, evidence-based policy roadmap to 2030

Drawing directly on the quantitative findings from this study, we propose a nuanced, context-specific policy roadmap. Unlike generic recommendations, the actions below are tied to specific coefficients, optimization results, and inequality decompositions from our analysis.

1. Financing: Differentiated investment targets by health system maturity
  - For countries with government health expenditure < 3% of GDP (e.g., many in sub-Saharan Africa): Increase public spending to at least 5% of GDP as a first priority. Our marginal effects (Figure 3) show that each $10 per capita increase yields a UHC SCI gain of 0.42 points—the highest return.
  - For countries with expenditure > 5% of GDP (e.g., upper-middle-income LMICs): Shift focus to reallocation. Our linear programming (Table 4) shows that moving 15% of budgets from tertiary care to primary care and digital health could raise UHC SCI by 9.7 points without additional funds.
2. Governance: Targeted anti-corruption and accountability metrics
  - The SEM (Table 1) attributes a 22% efficiency loss to corruption (range 18%–27%). We therefore recommend:
    - Mandatory open contracting for medicines and equipment in all LMICs with a Control of Corruption Index below −0.5.
    - Real-time expenditure dashboards linked to disbursement-pilot evidence from Rwanda and Georgia shows this reduces leakage by 12%–18% (46, 47).
    - Annual independent health sector audits published online, with civil society scorecards—a specific reform that improved governance in Kenya (8).
3. Digital health: Phased implementation by readiness
  - For countries with Digital Health Adoption Index (DHAI) < 40 (e.g., low-income countries): Start with mHealth for patients (ROI 4.1, Table 7) and telemedicine (readiness 72%).
  - For DHAI 40–60: Implement electronic health records and health information systems (ROI 2.9–3.2). Our trajectory analysis (Figure 8) shows that crossing DHAI = 40 accelerates UHC gains by 2.5× per year.
  - For DHAI > 60: Integrated digital platforms (coverage increase 7.3 points, Table 7) and AI decision support, but only after basic infrastructure is solid.
4. Equity: Pro-poor targeting using decomposition results
  - The Oaxaca-Blinder decomposition (Figure 7) shows that wealth (38.2%) and education (23.7%) are the main drivers of inequality. Therefore:
    - Conditional cash transfers for health targeted to the poorest two quintiles, specifically for maternal health (where concentration index is 0.192, Table 6).
    - Geographic weighted funding formulas that allocate 30% more to districts with highest poverty and lowest female education.
    - Zero-dose child campaigns (concentration index −0.156)—deploy community health workers to villages with zero-dose prevalence > 15%.
5. Resilience: Mandatory dual-function investments
  - Our resilience analysis (Table 8) identifies digital health infrastructure as the strongest determinant (reduces excess mortality by 21.5/100,000, contributes 25.3% to resilience). Therefore:
    - All primary care facilities must have offline-capable digital records that switch to emergency surveillance mode within 24 h.
    - Minimum resilience standards: IHR core capacity score > 70, health workforce density > 25/10,000, and at least one digital health application for real-time supply tracking. Countries below these thresholds should prioritize them in national health plans.
6. Regional tailoring (based on Figure 10):
  - Sub-Saharan Africa: Prioritize workforce (*β*=0.42) and governance (*β*=0.38). Example: double nurse graduation rates and implement district scorecards.
  - South Asia: Focus on financing reforms (*β*=0.48)—expand social health insurance to informal sector.
  - East Asia & Pacific: Leverage digital health (*β*=0.35)—scale national telemedicine networks.

These recommendations are not one-size-fits-all; they derive directly from the coefficients, elasticities, and optimization scenarios presented in Results sections 4.2–4.6. Policymakers can use Table 5 to select scenarios (e.g., Scenario B: reallocate 15% to primary care + digital) and Figure 5 to assess trade-offs between efficiency, equity, and resilience.

### 5.4 Limitations and mitigating strategies

This study has several important limitations. Where possible, we have implemented specific mitigating strategies, which are described below alongside each limitation.

1. Observational data and causality
  - *Limitation:* Despite using GMM and instrumental variables, residual confounding and reverse causality cannot be entirely ruled out.
  - *Mitigation applied:* We conducted placebo tests (using future health expenditure as a predictor of past outcomes)—no significant associations were found, supporting the directionality of our GMM specifications (see Online Supplementary Appendix Table 3). We also performed Hausman tests for endogeneity (48) and Sargan-Hansen overidentification tests (*p* > 0.10 for all models). These diagnostics increase confidence but do not prove causation.
2. Measurement uncertainty from irregular surveys
  - *Limitation:* The construction of a balanced annual panel from DHS/MICS (conducted every 3–5 years) introduces measurement error.
  - *Mitigation applied:* We used multiple imputation by chained equations (MICE) with 20 imputations, incorporating country and year fixed effects, GDP per capita, and regional averages as auxiliary variables. Sensitivity analyses comparing the imputed balanced panel with an unbalanced panel that excluded interpolated observations showed coefficients within ± 15% of main results (Online Supplementary Appendix Table 1). All main findings were robust to alternative imputation methods (linear interpolation only, no MICE).
3. Heterogeneity across LMICs
  - *Limitation:* Pooling 135 heterogeneous countries may mask important context-specific dynamics.
  - *Mitigation applied:* We conducted quantile regression (Figure 4) and regional sub-sample analyses (Figure 10) to explore variation. However, full stratification by income group or health system typology was not possible due to sample size constraints—we explicitly note this as a remaining limitation and a priority for future research.
4. Optimization assumptions
  - *Limitation:* Our optimization models assume a technocratic implementation environment with no political economy constraints, no institutional inertia, and full absorptive capacity.
  - *Mitigation applied:* We incorporated implementation constraints through scenario analysis (Table 5) and sensitivity testing (varying key parameters by ± 25%), which produced UHC SCI projections within ±4.2 points of main estimates. Stochastic programming (Figure 6) modeled budget uncertainty across 1,000 scenarios. We also present Pareto frontiers (Figure 5) that explicitly show trade-offs, rather than single optimal solutions. Readers are cautioned that real-world feasibility will be lower than these model-based upper bounds.
5. Projection linearity
  - *Limitation:* Projections to 2030 (Figure 11) assume that historical associations will continue linearly, which may not hold given nonlinear dynamics or emerging shocks.
  - *Mitigation applied:* Our projection model is a system dynamics model (45) that incorporates feedback loops and diminishing returns. We also ran worst-case and best-case scenarios (e.g., pandemic disruption, increased donor funding)—results are available in Online Supplementary Appendix Table 6. Nevertheless, all projections are illustrative, not predictions.
6. Unmeasured confounders
  - *Limitation:* There may be unmeasured factors (e.g., informal payments, social capital, climate events) that affect both health system inputs and outcomes.
  - *Mitigation applied:* We used country fixed effects to control for time-invariant unobserved heterogeneity, year fixed effects for common shocks, and region-time trends (interacted fixed effects) in robustness checks (Online Supplementary Appendix Table 4). Results were stable across specifications, reducing concerns about omitted variable bias.

In summary, while we cannot eliminate all limitations, the triangulation of econometric, optimization, and sensitivity methods, together with explicit mitigation strategies, strengthens the credibility of our findings. Remaining uncertainties are clearly communicated alongside each major estimate.

## 6 Conclusion

The path to Universal Health Coverage by 2030 is undeniably steep, but this study demonstrates that strategic, evidence-informed resource allocation could accelerate progress. Progress hinges not merely on the volume of resources but on the strategic orchestration of financing, governance, and technology (53). Our model-based scenarios suggest that reallocating existing resources toward primary care, governance, and digital transformation could yield substantial gains, though these estimates are illustrative and subject to uncertainty and implementation barriers. The COVID-19 pandemic has exposed the costs of fragmented health systems, but it has also illuminated a path forward: integrated, equitable, and digitally augmented health systems. However, achieving this vision requires not only technical optimization but also political will, institutional capacity, and context-specific adaptation. The era of incremental, siloed efforts is over. The time for bold, strategic health system transformation informed by evidence but realistic about constraints is now.

## Data Availability

The original contributions presented in the study are included in the article/Supplementary Material, further inquiries can be directed to the corresponding author.
