# Supplementary material for: Strengthening health systems in low and middle-income countries: performance determinants and pathways to universal health coverage
Source: Front Health Serv. 2026 Jun 16;6:1808077. doi: 10.3389/frhs.2026.1808077 (PMC13314607; doi:10.3389/frhs.2026.1808077)
Supplement: Supplementary file 1 [file Datasheet1.pdf]

ONLINE APPENDIX

Table of Contents

- **Appendix A:** Data Harmonization and Imputation Details
- **Table A1:** Sensitivity Analysis – Balanced vs. Unbalanced Panel
- **Table A2:** Robustness of Econometric Estimates to Alternative Imputation Methods
- **Table A3:** Placebo Tests – Future Health Expenditure as Predictor of Past Outcomes
- **Table A4:** Sensitivity of Optimization Results to Parameter Variations
- **Table A5:** Regional Sub-Analysis – Standardized Coefficients by WHO Region (Full Results)

Appendix A: Data Harmonization and Imputation Details

As described in Section 3.1, the Demographic and Health Surveys (DHS) and Multiple Indicator Cluster Surveys (MICS) are not conducted annually. To construct a balanced annual panel (2000–2023, 135 countries), we applied the following multi-step procedure:

1. **Linear interpolation** was applied for gaps between two survey rounds **only when the interval did not exceed 5 years**. For intervals longer than 5 years, no interpolation was performed (values treated as missing).
2. **Multiple imputation by chained equations (MICE)** was used for the remaining missing observations (approximately 18% of country-year observations for key UHC indicators). The imputation model included:
   - Country and year fixed effects
   - GDP per capita (log)
   - Regional averages (by WHO region)
   - Lagged values of the variable being imputed
   - 20 imputations were generated.
3. **Sensitivity analysis** (Table A1 below) compared results from the interpolated/imputed balanced panel with an **unbalanced panel** that excluded all interpolated observations (i.e., only original survey years retained).

TABLE A1: SENSITIVITY ANALYSIS – BALANCED VS. UNBALANCED PANEL

Coefficient comparison for key independent variables (dependent variable: UHC Service Coverage Index)

Variable	Balanced Panel (Main Model)	Unbalanced Panel (No Interpolation)	Difference (%)
Government Health Expenditure per capita (log)	2.64*** (0.76)	2.51*** (0.81)	-4.9%
Out-of-pocket Health Expenditure (% of THE)	-0.18*** (0.05)	-0.19*** (0.06)	+5.6%
Physician Density (log)	2.31** (1.04)	2.18** (1.09)	-5.6%
Government Effectiveness Index	3.61*** (1.15)	3.48*** (1.21)	-3.6%
Digital Health Adoption Index	0.25*** (0.07)	0.23*** (0.08)	-8.0%

Notes: Robust standard errors clustered at country level in parentheses. *** p<0.01, ** p<0.05. THE = Total Health Expenditure. All coefficients within ±15% of main estimates, supporting robustness.

TABLE A2: ROBUSTNESS OF ECONOMETRIC ESTIMATES TO ALTERNATIVE IMPUTATION METHODS

Fixed effects coefficients (UHC SCI as dependent variable) under different missing data treatments

Variable	MICE (Main)	Linear Interpolation Only (No MICE)	Listwise Deletion (Complete Cases Only)
Government Health Expenditure per capita (log)	2.64*** (0.76)	2.58*** (0.79)	2.44*** (0.84)
Out-of-pocket Health Expenditure (% of THE)	-0.18*** (0.05)	-0.19*** (0.05)	-0.20*** (0.06)
Physician Density (log)	2.31** (1.04)	2.25** (1.07)	2.12** (1.11)
Government Effectiveness Index	3.61*** (1.15)	3.55*** (1.18)	3.42*** (1.22)
Digital Health Adoption Index	0.25*** (0.07)	0.24*** (0.08)	0.22*** (0.08)
Observations (N)	3,240	3,240	2,684

Notes: All models include country and year fixed effects. Standard errors clustered at country level. No substantive conclusions change across methods.

TABLE A3: PLACEBO TESTS – FUTURE HEALTH EXPENDITURE AS PREDICTOR OF PAST OUTCOMES

Testing for reverse causality: using 3-year ahead health expenditure to predict current UHC SCI

Predictor (Lead)	Coefficient	Standard Error	P-value	Interpretation
Government Health Expenditure (t+1)	0.31	0.48	0.52	Not significant
Government Health Expenditure (t+2)	0.24	0.52	0.64	Not significant
Government Health Expenditure (t+3)	0.18	0.55	0.74	Not significant

Notes: Fixed effects regression with current UHC SCI as dependent variable. Lack of significant associations supports the directionality assumed in GMM models (past expenditure → current outcomes, not reverse).

TABLE A4: SENSITIVITY OF OPTIMIZATION RESULTS TO PARAMETER VARIATIONS

Impact of varying key model parameters by ±25% on estimated UHC SCI gain (Scenario C: 20% reallocation)

Parameter varied	Variation	UHC SCI Gain (points)	Change from main estimate
Main estimate (no variation)	–	12.4	–
Elasticity of UHC to primary care spending	+25%	14.8	+2.4
Elasticity of UHC to primary care spending	-25%	10.1	-2.3
Discount rate (future health gains)	+25% (higher discount)	11.2	-1.2
Discount rate (future health gains)	-25% (lower discount)	13.5	+1.1
Implementation lag (years to full effect)	+50% (slower)	10.8	-1.6
Implementation lag (years to full effect)	-50% (faster)	14.2	+1.8
Absorptive capacity constraint	+25% (more capacity)	13.9	+1.5
Absorptive capacity constraint	-25% (less capacity)	10.6	-1.8

Notes: All variations produce UHC SCI gains within ±2.4 points of the main estimate (range: 10.1–14.8). The main conclusion that reallocation yields substantial gains remains robust.

TABLE A5: REGIONAL SUB-ANALYSIS – STANDARDIZED COEFFICIENTS BY WHO REGION (FULL RESULTS)

Dependent variable: UHC Service Coverage Index. Fixed effects with full controls.

WHO Region	Health Financing	Health Workforce	Governance	Digital Health	Service Delivery	R-squared
Sub-Saharan Africa	0.31*** (0.08)	0.42*** (0.10)	0.38*** (0.09)	0.19** (0.07)	0.24** (0.09)	0.72
South Asia	0.48*** (0.11)	0.21* (0.12)	0.18* (0.10)	0.22** (0.09)	0.19* (0.10)	0.68
East Asia & Pacific	0.29*** (0.09)	0.27** (0.11)	0.32*** (0.08)	0.35*** (0.09)	0.21** (0.08)	0.74
Latin America & Caribbean	0.35*** (0.08)	0.29** (0.10)	0.31*** (0.07)	0.26** (0.09)	0.28*** (0.08)	0.70
Middle East & North Africa	0.27** (0.10)	0.23* (0.12)	0.29** (0.09)	0.24** (0.10)	0.31*** (0.09)	0.66
Europe & Central Asia	0.33*** (0.09)	0.18* (0.10)	0.45*** (0.11)	0.27** (0.08)	0.22** (0.09)	0.69

Notes: Standard errors in parentheses. *** $p<0.01$, ** $p<0.05$, * $p<0.1$. Coefficients are standardized. The table extends Figure 9 by providing full numerical estimates and standard errors.

ADDITIONAL METHODOLOGICAL NOTES (ONLINE ONLY)

Software and Code

- All econometric analyses were performed in **Stata 18.0** using the following commands: xtreg, fe, xtabond2, sem, qreg.
- Optimization models were implemented in **MATLAB R2023b** using the linprog, fgoalattain, and custom stochastic programming routines (sample average approximation with 1,000 scenarios).
- Structural equation models were estimated in **Mplus 8.8** using maximum likelihood with robust standard errors.
- Figures were generated in **R 4.3.0** using ggplot2 and plotly.

Replication Package

A full replication package including:

- The imputed balanced panel dataset (CSV format)
- Do-files for Stata (all econometric models)
- MATLAB scripts for optimization
- R scripts for figures

will be made available at https://github.com/[repository]/lmic_uhc_optimization upon publication. Interim requests can be directed to the corresponding author.
